# White Fluorescent Organic Light-Emitting Diodes with 100% Power Conversion

**DOI:** 10.34133/research.0009

**Published:** 2022-12-19

**Authors:** Dongxue Ding, Zicheng Wang, Chunbo Duan, Chunmiao Han, Jing Zhang, Shuo Chen, Ying Wei, Hui Xu

**Affiliations:** Key Laboratory of Functional Inorganic Material Chemistry, Ministry of Education, School of Chemistry and Materials, Heilongjiang University, Harbin, Heilongjiang, China.

## Abstract

Energy-efficient lighting sources are desired to provide another solution of carbon emission reduction. White organic light-emitting diodes are promising, because of theoretical internal quantum efficiencies for 100% electric-to-light conversion. However, pure organic fluorescent materials still face a challenge in harvesting triplet excitons for radiation. Herein, we report a white fluorescent organic light-emitting diode having an external quantum efficiency of 30.7% and a power efficiency of 120.2 lm W^−1^. In the single emissive layers, we use blue thermally activated delayed fluorescent emitters to sensitize a yellow fluorescent emitter. Transient photoluminescence and electroluminescence analyses suggest that a blue thermally activated delayed fluorescent molecule with ~100% reverse intersystem crossing efficiency and negligible triplet nonradiative rate constant completely converts triplet to singlet, suppressing triplet quenching by a yellow fluorescent emitter and ensuring 100% power conversion.

## Introduction

White organic light-emitting diodes (OLEDs) based on pure organic materials are promising as highly efficient large-area lighting sources, because of the merits of low cost, environmental sustainability, and large-scale production [1,2]. However, according to spin statistics of electrogenerated excitons, organic fluorophores can use only 25% singlet excitons [3]. In contrast, phosphorescent organometallic complexes of noble metals can utilize 100% excitons of both singlet and triplet, through intersystem crossing (ISC) [4]. Recently, thermally activated delayed fluorescence (TADF) has emerged as a competitive alternative for harvesting 100% excitons for electroluminescence (EL) [5,6]. Most of TADF molecules are pure organic, featuring donor–acceptor structures and near-zero singlet–triplet energy gaps [7,8]. In this case, reverse intersystem crossing (RISC) can convert the nonradiative triplet of the TADF molecule to a radiative singlet, leading to 100% exciton harvesting [9–11]. Monochromic TADF diodes have realized internal quantum efficiency (IQE, *η*_IQE_) and external quantum efficiency (EQE, *η*_EQE_) respectively approaching 100% and 30%, which are already comparable to the values of state-of-the-art phosphorescent OLEDs [12–16]. Despite the high singlet radiative rate and feasible RISC, the triplet is still dominant in the singlet–triplet equilibrium, because of the kinetic advantage of exothermic ISC to endothermic RISC [17]. Thus, TADF emitters suffer from both triplet–triplet annihilation and singlet–triplet quenching [18], inducing more marked efficiency roll-offs of TADF diodes than phosphorescent counterparts [19–21]. Due to the collisional nature of quenching effects, TADF molecules are highly sensitive to intermolecular interactions, especially for blue emitters [22–25]. Although there are efficient TADF-phosphorescence hybrid [26–28] and full-TADF white OLEDs [29–31] reported, the interactions between blue and green/yellow/red TADF emitters are quite complicated [32], requiring accurate optimization of molecular configurations and device structures [2,33,34].

**Fig. 1.**
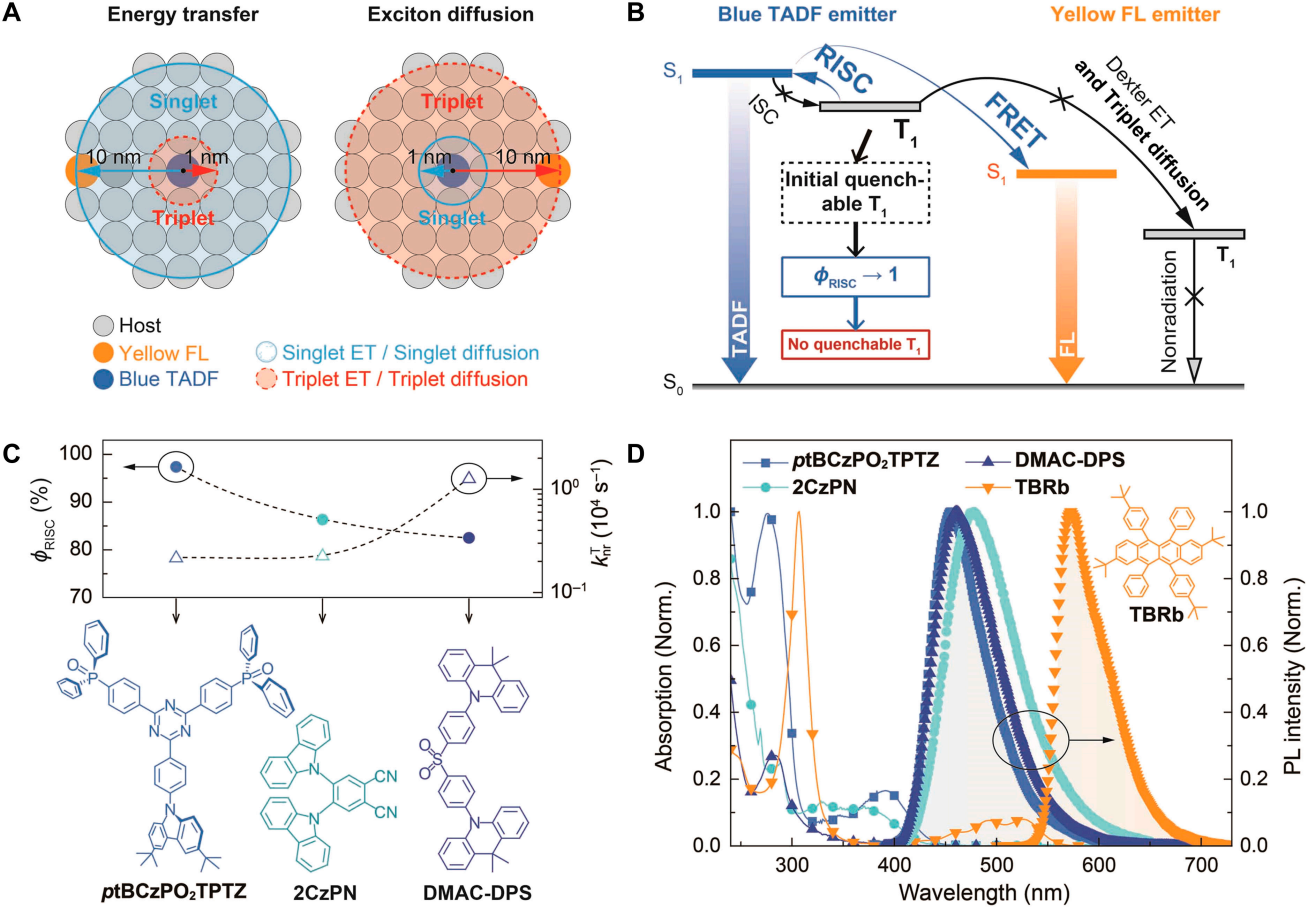
Design strategy of hyperfluorescence white-emitting systems. (A) Spatial features of triplet energy transfer and diffusion in a white hyperfluorescence system consisting of a yellow FL emitter, a blue TADF sensitizer, and a host matrix. (B) Key transitions and energy transfer processes between the blue TADF sensitizer and the yellow FL emitter. (C) Chemical structures of 9-(4-(4,6-Bis(4-(diphenylphosphoryl)phenyl)-1,3,5-triazin-2-yl)phenyl)-3,6-di-*tert*-butyl-carbazole (*p*tBCzPO_2_TPTZ), 4,5-bis(carbazol-9-yl)-1,2-dicyanobenzene (2CzPN), and Bis[4-(9,9-dimethyl-9,10-dihydroacridine)phenyl]sulfone (DMAC-DPS), and RISC efficiencies (*ϕ*_RISC_) and triplet nonradiative rate constants ($k_{\mathrm{nr}}^{T}$) of their 40% doped 4,6-bis(diphenylphosphoryl)dibenzofuran (DBFDPO) films (vacuum evaporated, 100 nm). (D) Electronic absorption spectra of *p*tBCzPO_2_TPTZ, 2CzPN, DMAC-DPS, and 2,8-di-*tert*-butyl-5,11-bis(4-*tert*-butylphenyl)-6,12-diphenyltetracene (TBRb) in dichloromethane (10^-6^ mol l^−1^) and photoluminescence (PL) spectra of *p*tBCzPO_2_TPTZ, 2CzPN, DMAC-DPS, and TBRb doped in DBFDPO films (40%, vacuum evaporated, 100 nm). Inset shows the chemical structure of TBRb.

Their long-lived feature makes triplets inherently easy to be quenched by intermolecular interactions. Therefore, enhancing triplet-to-singlet conversion is a fundamental way to restrain quenching-induced efficiency reduction and roll-off [35–37]. It is demonstrated that TADF molecules can be used as triplet sensitizers of traditional fluorescent (FL) emitters [38,39]. The so-called “hyperfluorescence” diodes with emissive layers containing FL and TADF molecules can also achieve *η*_EQE_ more than 20%. In these devices, singlet excitons converted from the first triplet excited state (T_1_) of TADF sensitizers can be subsequently captured and utilized by FL emitters, through Förster resonance energy transfer (FRET). This process not only makes singlet–triplet equilibrium become more favorable to singlet formation but also transforms relatively labile charge transfer excitons of TADF molecules into stable Frenkel excitons of FL emitters. It is beneficial to comprehensively mitigate nonradiative deactivation of excitons [3]. However, triplet energy transfer from TADF sensitizers to the dark T_1_ state of FL emitters should be thoroughly avoided. Dexter energy transfer (DET) of triplets is based on short-distance charge exchange (within 1 nm). Thus, low doping concentration of FL emitters is believed to be necessary and sufficient to maintain a long enough average distance between TADF and FL molecules and thereby prevent triplet DET (Fig. 1A). However, at present, hyperfluorescence OLEDs hardly achieved *η*_EQE_ reaching the state-of-the-art value of TADF diodes (30%). It means that a part of excitons was still wasted during the energy transfer process [40,41].

Charge transfer excitons of TADF molecules are much easier to long-range delocalize, compared to Frenkel excitons. Besides DET, another feature of triplet excitons must be noted: triplet diffusion distance (≥10 nm) is at least one order of magnitude larger than singlet diffusion distance (≤1 nm) [42] (Fig. 1A). In this case, triplet diffusion actually provides another channel for triplet capture by FL emitters. Triplet DET and triplet diffusion are respectively dominant at relatively high and low doping concentration. The energy level relationship between TADF sensitizers and FL emitters shows that it is difficult to completely eradicate triplet migration between their T_1_ states, since effective FRET should be based on a suitable concentration of FL emitters (Fig. 1B). Therefore, an “ideal” situation is triplet-involved processes confined on TADF molecules, which requires the following: (a) the TADF sensitizer completely converts triplet to singlet, and (b) before conversion, triplet nonradiation is effectively suppressed. As a proof of concept, in this contribution, we demonstrate a white hyperfluorescence system featuring triplet-free exciton allocation. Three blue TADF emitters named *p*tBCzPO_2_TPTZ, 2CzPN, and DMAC-DPS are respectively used as blue-emitting sensitizers to fabricate hyperfluorescence white OLEDs with a conventional yellow FL emitter TBRb (Fig. 1C). These 3 molecules have similar molecular polarities, thus excluding the influence of dipole–dipole interactions between TADF and FL emitters. In the 4,6-bis(diphenylphosphoryl)dibenzofuran (DBFDPO) matrix with a doping concentration of 40% for weight percentage, the RISC efficiency (*ϕ*_RISC_) and triplet nonradiative rate constant ($\;k_{\mathrm{nr}}^{T}$) of *p*tBCzPO_2_TPTZ are 97% and 2.15 × 10^3^ s^−1^, respectively. In contrast, *ϕ*_RISC_ values of 2CzPN and DMAC-DPS (~80%) are much lower, while the $k_{\mathrm{nr}}^{T}$ value of the 2CzPN-based film (2.24 × 10^3^ s^−1^) is equal to that of *p*tBCzPO_2_TPTZ, but is only one-fifth of that of the DMAC-DPS-based film (1.26 × 10^4^ s^−1^). The orthogonal correlation of *ϕ*_RISC_ and $k_{\mathrm{nr}}^{T}$ values for these molecules establishes the basis for figuring out exciton utilization and quenching processes and the relative structure–property relationships. Blue emissions from *p*tBCzPO_2_TPTZ, 2CzPN, and DMAC-DPS are complementary to the yellow emission of TBRb, and simultaneously overlap with the *π*→π* absorption band of TBRb in a large range of 400 to 550 nm, leading to efficient FRET (Fig. 1D). Compared to 2CzPN and DMAC-DPS, *p*tBCzPO_2_TPTZ with ~100% *ϕ*_RISC_ and negligible $k_{\mathrm{nr}}^{T}$ effectively mitigates triplet quenching by TBRb. As a result, warm-white OLED of DBFDPO:40% *p*tBCzPO_2_TPTZ:0.1% TBRb achieved a state-of-the-art *η*_EQE_ of up to 30.7%, corresponding to 100% *η*_IQE_, and a record-high power efficiency (*η*_PE_) of 120.2 lm W^−1^.

## Results

### Steady-state photophysical properties

**Fig. 2.**
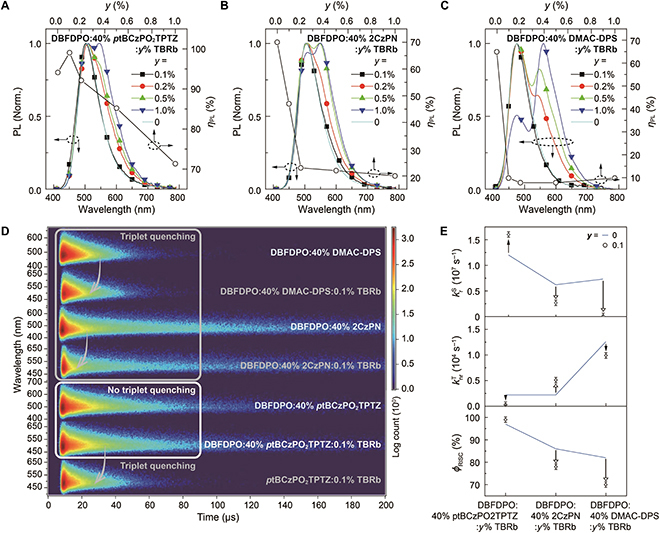
Photophysical properties of white hyperfluorescence films. PL spectra of DBFDPO:40% *p*tBCzPO_2_TPTZ:*y*% TBRb (A), DBFDPO:40% 2CzPN:*y*% TBRb (B), and DBFDPO:40% DMAC-DPS:*y*% TBRb (C) films (vacuum evaporated, 100 nm; *y* = 0 to 1.0%). (D) Time-resolved emission spectra of DBFDPO:40% blue TADF emitters:*y*% TBRb and *p*tBCzPO_2_TPTZ:0.1% TBRb films. Blue TADF emitters are *p*tBCzPO_2_TPTZ, 2CzPN, and DMAC-DPS, respectively. (E) TADF parameter variation after TBRb doping. $k_{r}^{S}$ is singlet radiative rate constant. *y* = 0 for blue or 0.1 for white.

The photoluminescence (PL) quantum yield (*η*_PL_) of the DBFDPO:40% *p*tBCzPO_2_TPTZ film reaches 94%, which is higher than ~70% of 2CzPN- and DMAC-DPS-doped films (Fig. 2A to C). More importantly, for DBFDPO:*x*% *p*tBCzPO_2_TPTZ:*y*% TBRb dually doped films, at *x* = 40, a slight codoping of 0.1% TBRb largely increases *η*_PL_ to 99%, reflecting nearly unitary radiative efficiency and completely suppressed nonradiative transitions. However, further increasing TBRb concentration gradually decreases *η*_PL_. Similarly, when *x* ≤ 40, the *η*_PL_ of DBFDPO:*x*% *p*tBCzPO_2_TPTZ:0.1% TBRb films gradually increases and reaches the maximum at *x* = 40 (Fig. S1). Further increasing *x* also induces *η*_PL_ decrease. The *η*_PL_ inflexion is a result of a delicate balance between exciton radiation and quenching. In contrast, for DBFDPO:40% 2CzPN:*y*% TBRb films, *η*_PL_ decreases to 47% even at *y* = 0.1, and further halved to ~20% at *y* ≥ 0.2. The quenching effect of TBRb is the worst for DBFDPO:40% DMAC-DPS:*y*% TBRb films, whose *η*_PL_ is as low as ~10% when *y* ≥ 0.1. Notably, *p*tBCzPO_2_TPTZ-based films reveal gradually reduced *η*_PL_ inversely proportional to *x* and *y*, reflecting a linear dependence of triplet quenching on *p*tBCzPO_2_TPTZ–TBRb distance. On the contrary, the *η*_PL_ of 2CzPN- and DMAC-DPS-based films sharply decreases to the minimum values at quite low *y*, especially for the latter, indicating the dominance of relatively distance-insensitive triplet diffusion in emission quenching. It shows that *η*_PL_ variation is directly related to *ϕ*_RISC_ of these blue TADF emitters.

**Fig. 3.**
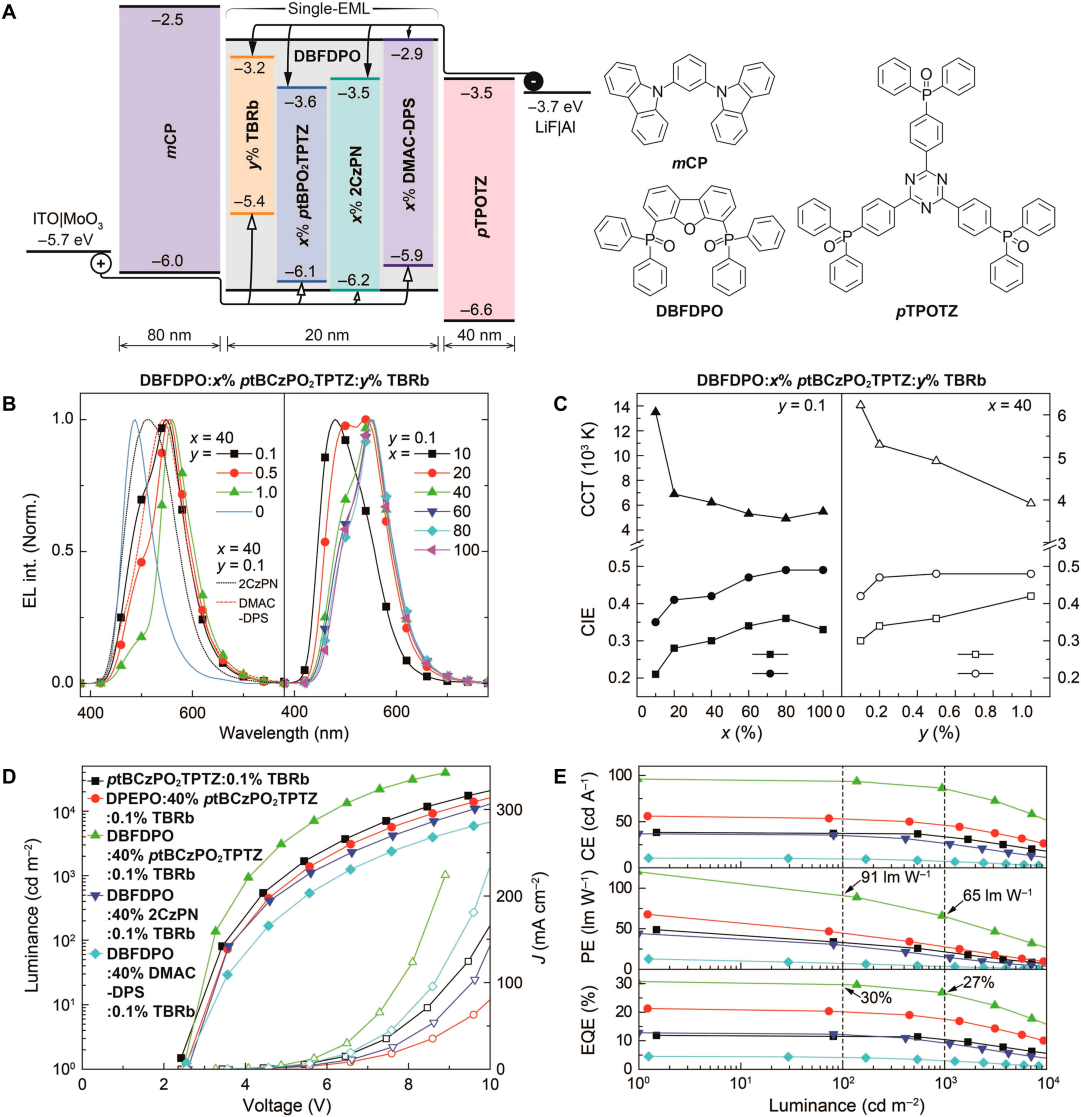
OLED performance. (A) Device structure and chemical structures of used materials. (B) Electroluminescence (EL) spectra at 1,000 nits. (C) Commission Internationale de l'Eclairage (CIE) coordinates and correlated color temperature (CCT) values under different doping concentrations at 1,000 nits. (D) Luminance–voltage–current density (*J*) relationships. (E) Efficiency–luminance correlations. CE, PE, and EQE refer to current efficiency, power efficiency, and external quantum efficiency, respectively. PE and EQE values at 100 and 1,000 cd m^−2^ were highlighted with arrows.

At extremely low TBRb concentration (*y* = 0.1), the yellow emission intensities are independent of blue TADF emitters (Fig. 2A to C and Fig. S2). The ~100% *η*_PL_ of the DBFDPO:40% *p*tBCzPO_2_TPTZ:0.1% TBRb film is a combined result of 100% FRET efficiency, RISC enhancement, and triplet quenching suppression. In contrast, 2CzPN- and DMAC-DPS-based films show slightly changed PL spectra with sharply decreased *η*_PL_, reflecting exciton allocation-induced triplet nonradiation. Since triplet diffusion rather than triplet DET is predominant at *y* = 0.1, it suggests that triplet diffusion-induced quenching is negligible for *p*tBCzPO_2_TPTZ-based films, but significant in 2CzPN- and DMAC-DPS-based films. Furthermore, along with *y* increasing, yellow emissions of DMAC-DPS-based films rapidly increase, which are largely stronger than those of 2CzPN-based films, while *p*tBCzPO_2_TPTZ-based films display the weakest yellow emissions. Since FRET is a dipole–dipole resonance-based long-range energy transfer, these 3 blue TADF emitters are comparable in the FRET process. The large PL spectra difference of the dually doped films is mainly caused by intermolecular interaction-based exciton/charge exchange, namely, singlet DET. The combined analysis of *η*_PL_ and PL variations suggests that *η*_PL_ and yellow intensity are simultaneously related to the intermolecular interaction intensity in the dually doped films. Symmetrically bar-shaped DMAC-DPS has the strongest intermolecular interactions. At the same time, the highest occupied molecule orbital and lowest unoccupied molecule orbital energy levels of TBRb are respectively shallower and deeper than those of DMAC-DPS, leading to direct charge/exciton capture by TBRb (Fig. 3A). Consequently, singlet and triplet diffusions in DMAC-DPS-based films are the strongest, giving rise to the most marked yellow emissions and the lowest *η*_PL_. On the contrary, the asymmetric and sphere-like configuration of 2CzPN somewhat reduces intermolecular interactions and limits singlet/triplet diffusions and DET, rendering the weaker yellow emissions but the higher *η*_PL_ values of 2CzPN-based films, which is similar to our previously reported all-TADF systems [29]. In comparison, *p*tBCzPO_2_TPTZ with large steric hindrance further restrains intermolecular interactions and singlet/triplet diffusion in its films. Triplet DET becomes the main channel of emission quenching, which is also limited but still appreciable at high *x* and *y*. Moreover, for *p*tBCzPO_2_TPTZ-based films, compared to DBFDPO, another conventional host, bis{2-[di(phenyl)phosphino]-phenyl}ether oxide (DPEPO), with a larger steric hindrance further decreases yellow emission intensity and mitigates *η*_PL_ reduction at high *y* (Fig. S3). On the contrary, without host, *η*_PL_ reduction is accelerated (Fig. S4).

### Time-resolved emission properties

Transient emission spectra (TRES) show that compared to blue-emitting 2CzPN and DMAC-DPS films, 0.1% TBRb induces marked delayed fluorescence (DF) quenching (Fig. 2D), and DF intensities and lifetimes are approximately independent of TBRb concentration (0.1% to 1.0%) (Fig. S5). This result suggests that DF quenching in 2CzPN and DMAC-DPS films is primarily due to triplet diffusion, which is consistent with their *η*_PL_ variations. In contrast, the smallest $k_{\mathrm{nr}}^{T}$ makes the triplet state of *p*tBCzPO_2_TPTZ much less sensitive to TBRb doping; therefore, TRES contours of DBFDPO:*x*% *p*tBCzPO_2_TPTZ:*y*% TBRb films (*x* = 40%; *y* = 0 and 0.1) are nearly identical, despite the gradual DF reduction at *y* > 0.1 (Fig. S6). In the DPEPO matrix, the DF variation of *p*tBCzPO_2_TPTZ is similar (Fig. S7). At *y* = 0.1, when *x* ≤ 40, DF intensities and lifetimes remain stable, but prompt fluorescence (PF) intensity is directly proportional to *x*. It means that in this *x* range, triplet quenching can be effectively suppressed, while increasing *x* reduces *p*tBCzPO_2_TPTZ–TBRb distance, thus enhancing FRET. At an *x* range of 40 to 100, different to slightly changed PF, DF intensity and lifetime sharply decrease from *x* = 40 to 60 and further gradually decline until *x* = 100. Actually, at *x* = 100, the PF and DF properties of the binary films are independent on *y*. Therefore, when *p*tBCzPO_2_TPTZ becomes the majority (*x* > 50), DET via direct interactions between *p*tBCzPO_2_TPTZ and TBRb is still inevitable, rendering triplet quenching. Nevertheless, the critical *x*% reaching 40% still demonstrates that ~100% *ϕ*_RISC_ and the largely reduced $k_{\mathrm{nr}}^{T}$ of *p*tBCzPO_2_TPTZ indeed effectively alleviate triplet quenching by TBRb, which establishes the feasibility of balancing FRET and triplet exclusion for optimal exciton allocation.

PF time decay curves of singly doped blue-emitting films are nearly identical to those of blue PF emissions from 0.1% TBRb codoped films (Fig. 2D and Fig. S8A). Time decay curves of yellow PF and DF are consistent with those of the corresponding blue PF and DF, respectively. PF lifetimes of TBRb in dually doped films are markedly larger than those of the DBFDPO:5% TBRb film (Fig. S8B). Therefore, yellow emissions of TBRb in these films are mainly ascribed to FRET from blue TADF emitters. However, 0.1% TBRb significantly shortens blue DF lifetimes of 2CzPN/DMAC-DPS-based films. In contrast, for DBFDPO:*x*% *p*tBCzPO_2_TPTZ:*y*% TBRb films, at *x* = 40, blue DF time decay curves overlap for *y* = 0 and 0.1, owing to effectively suppressed triplet diffusion (Fig. S9). Further increasing *y* of *p*tBCzPO_2_TPTZ-based films hardly changes blue PF lifetimes, but gradually reduces blue DF lifetimes, reflecting intensified DET-induced triplet quenching. On the other hand, at *y* = 0.1, increasing *x* from 10 to 40 induces elongated blue PF lifetimes of *p*tBCzPO_2_TPTZ, but does not change its blue DF lifetimes (Fig. S10). In this *x* range, direct excitation of *p*tBCzPO_2_TPTZ’s S_1_ state is gradually enhanced, but triplet quenching can still be effectively controlled. However, further increasing *x* from 40 to 100, blue PF lifetime becomes stable, but blue DF lifetime gradually declines, reflecting marked triplet quenching. At *x* = 100, increasing *y* from 0.1 to 1.0 can still shorten blue DF lifetime, but the variation is much smaller than at *x* = 40. These results further suggest that for DBFDPO:*x*% *p*tBCzPO_2_TPTZ:*y*% TBRb films, *x* = 40 is the critical point of balancing FRET and triplet quenching, and only a considerable *x* or *y* increase leads to serious triplet quenching, indicating the predominance of triplet DET in the quenching process.

Compared to the DBFDPO:40% *p*tBCzPO_2_TPTZ film, the *ϕ*_RISC_ of the DBFDPO:40% *p*tBCzPO_2_TPTZ:0.1% TBRb film is further improved to 99%, and in particular, its $k_{\mathrm{nr}}^{T}$ largely decreases by one order of magnitude to 3.0 × 10^2^ s^−1^ (Fig. 2E and Table S1). Simultaneously, its singlet radiative rate constant ($k_{r}^{S}$) significantly increases by one-third to 1.6 × 10^7^ s^−1^. Consequently, 0.1% TBRb facilitates RISC and singlet radiation and mitigates triplet quenching in *p*tBCzPO_2_TPTZ-based films. In this case, triplet excitons can be completely converted to radiative singlets by *p*tBCzPO_2_TPTZ and then respectively allocated to itself and TBRb, resulting in 100% triplet harvesting. In contrast, 0.1% TBRb induces sharp *ϕ*_RISC_ decreases of 8% and 12% for 2CzPN- and DMAC-DPS-based films, respectively. Furthermore, the $k_{\mathrm{nr}}^{T}$ of the 2CzPN-based film is doubled, but its $k_{r}^{S}$ is halved, in addition to 13% increased $k_{\mathrm{nr}}^{S}$. Although the $k_{\mathrm{nr}}^{T}$ and $k_{\mathrm{nr}}^{S}$ of the DMAC-DPS-based film are reduced by 0.1% TBRb doping, its $k_{r}^{S}$ decreased by 70-fold to 1.0 × 10^5^ s^−1^, which was ^1^/_10_ of its $k_{\mathrm{nr}}^{S}$. Therefore, TBRb doping hinders RISC and aggravates quenching effects in 2CzPN- and DMAC-DPS-based films. These results show that in these dually doped films, compared to ~70% *ϕ*_RISC_ of 2CzPN and DMAC-DPS, *p*tBCzPO_2_TPTZ with ~100% *ϕ*_RISC_ and small $k_{\mathrm{nr}}^{T}$ establishes a basis for TBRb to largely enhance radiative transitions and reduce nonradiation. Notably, the TADF parameters of the *p*tBCzPO_2_TPTZ:0.1% TBRb film are comparable to those of the DBFDPO:40% 2CzPN:0.1% TBRb film. It suggests that despite effectively limited triplet diffusion, triplet DET to FL dopants can still cause serious quenching of TADF sensitizers.

### EL performance

White OLEDs were fabricated by vacuum evaporation, following the simple 3-layer architecture (Fig. 3A). Single emissive layers DBFDPO:*x*% blue TADF emitter:*y*% TBRb were employed, in addition to using *N*,*N*′-dicarbazolyl-3,5-benzene and 2,4,6-Tris(4-(diphenylphosphoryl)phenyl)-1,3,5-triazine as hole and electron transporting layers, respectively. Concentrations of *x* and *y* were carefully explored to determine the optimal parameters (Figs. S11 to S16 and Table S2). Simultaneously, in accord with PL spectra, increasing *x* and *y* enhanced yellow emissions in EL spectra, as a result of FRET improvement. Compared to 2CzPN and DMAC-DPS with stronger intermolecular interactions, *p*tBCzPO_2_TPTZ-based devices displayed dual-peak complementary white emissions with largely higher color purities (Fig. 3B). It is noted that at *y* = 0.1, when *x* ≥ 60, the EL spectra of *p*tBCzPO_2_TPTZ-based devices almost overlapped. It indicates that at *x* ≥ 60, the FRET process tended to be stable, and triplet DET became dominant, leading to unchanged exciton allocation ratios for blue and yellow emissions. In contrast, at *x* = 40, along with *y* increasing from 0.1 to 1.0, EL correlated color temperature changed from 6,225 K (*y* = 0.1), 4,914 K (*y* = 0.5), to 3,921 K (*y* = 1.0) (Fig. 3C and Fig. S17), which are respectively close to correlated color temperature values of standard illuminants D65 (6,500 K, artificial daylight), D50 (5,000 K, simulated sunlight), and A (2,856 K, incandescent light).

It is shown that at *x* = 40, *p*tBCzPO_2_TPTZ-based devices realized the best performances (Figs. S11 and S14). In the *y* range of 0.1 to 1.0, besides luminescence beyond 30,000 cd m^−2^, the driving voltages at 1, 100, and 1,000 cd m^−2^ were as low as ~2.5, ~3.3, and ~4.1 V, respectively (Fig. 3D and Fig. S14), which are the lowest values reported so far among white hyperfluorescence OLEDs [37,43] and also comparable to the best results from all kinds of white EL devices [44] (Table S3). Moreover, the driving voltages were roughly inversely proportional to *x*. Obviously, at high doping concentrations, *p*tBCzPO_2_TPTZ with ambipolar feature made significant contributions to carrier injection and transportation. 2CzPN- and DMAC-DPS-based devices revealed similar situations (Figs. SS12 and S13). Nevertheless, *p*tBCzPO_2_TPTZ-based host-free devices (*x* = 100) displayed slightly larger driving voltages than those of DBFDPO hosted analogs. Moreover, the incorporation of DPEPO with worse electrical properties increased driving voltages. Therefore, the electrical performance of the host matrix is also considerable for balancing carrier flux, while quenching suppression by the host matrix improves luminance at specific current density (*J*), thereby, in turn, reducing driving voltages. Furthermore, the incorporation of TBRb largely increased the maximum luminance, since FRET to TBRb facilitates RISC and restrains triplet concentration quenching.

For DBFDPO:*x*% *p*tBCzPO_2_TPTZ:*y*% TBRb-based devices, at *y* = 0.1, the maximum *η*_EQE_ markedly increased from 9.6% at *x* = 10 to the highest value of 30.7% at *x* = 40 and then decreased to 11.8% at *x* = 100 (Figs. S11 and S15). Using the DPEPO matrix can still achieve the maximum *η*_EQE_ at *x* = 40 but reduces *η*_EQE_ decrease at *x* = 60 (Fig. S16). The optimal *x* for 2CzPN-based devices was also 40 (Fig. S12), different to that (10) for DMAC-DPS-based analogs (Fig. S13). At *x* = 40, the maximum *η*_EQE_ of *p*tBCzPO_2_TPTZ-based devices was inversely proportional to *y*, decreasing to 21.6% at *y* = 1.0. Thus, the key factor inducing *η*_EQE_ reduction is triplet quenching, including blue TADF emitter-based triplet–triplet and singlet–triplet annihilations and TBRb-induced triplet quenching. Nevertheless, *η*_EQE_ was more sensitive to *x*, reflecting the predominance of *p*tBCzPO_2_TPTZ in exciton utilization (Fig. S18). At *x* =40 and *y* = 0.1, the maximum *η*_EQE_ of *p*tBCzPO_2_TPTZ-based white OLEDs (30.7%) was 2.5-fold of that of 2CzPN-based devices (12.4%) and 6.3-fold of that of DMAC-DPS-based analogs (4.9%) (Fig. 3E). *η*_EQE_ values of *p*tBCzPO_2_TPTZ:*y*% TBRb-based devices were nearly equal to those of 2CzPN (*x* = 40)-based ternary devices. These results suggest that triplet DET and diffusion to TBRb are the main channels of triplet quenching. Owing to low driving voltages, *p*tBCzPO_2_TPTZ endowed its white hyperfluorescence OLEDs with a new record *η*_PE_ of 120.2 lm W^−1^ for the maximum. At 100 and 1,000 nits, *η*_PE_ remained 91.1 and 65.3 lm W^−1^, corresponding to *η*_EQE_ values of 30.0% and 26.9%, respectively. These efficiency values are far beyond previous reports of white hyperfluorescence OLEDs [37,43] and also superior to all kinds of white EL devices [44] (Table S3).

**Fig. 4.**
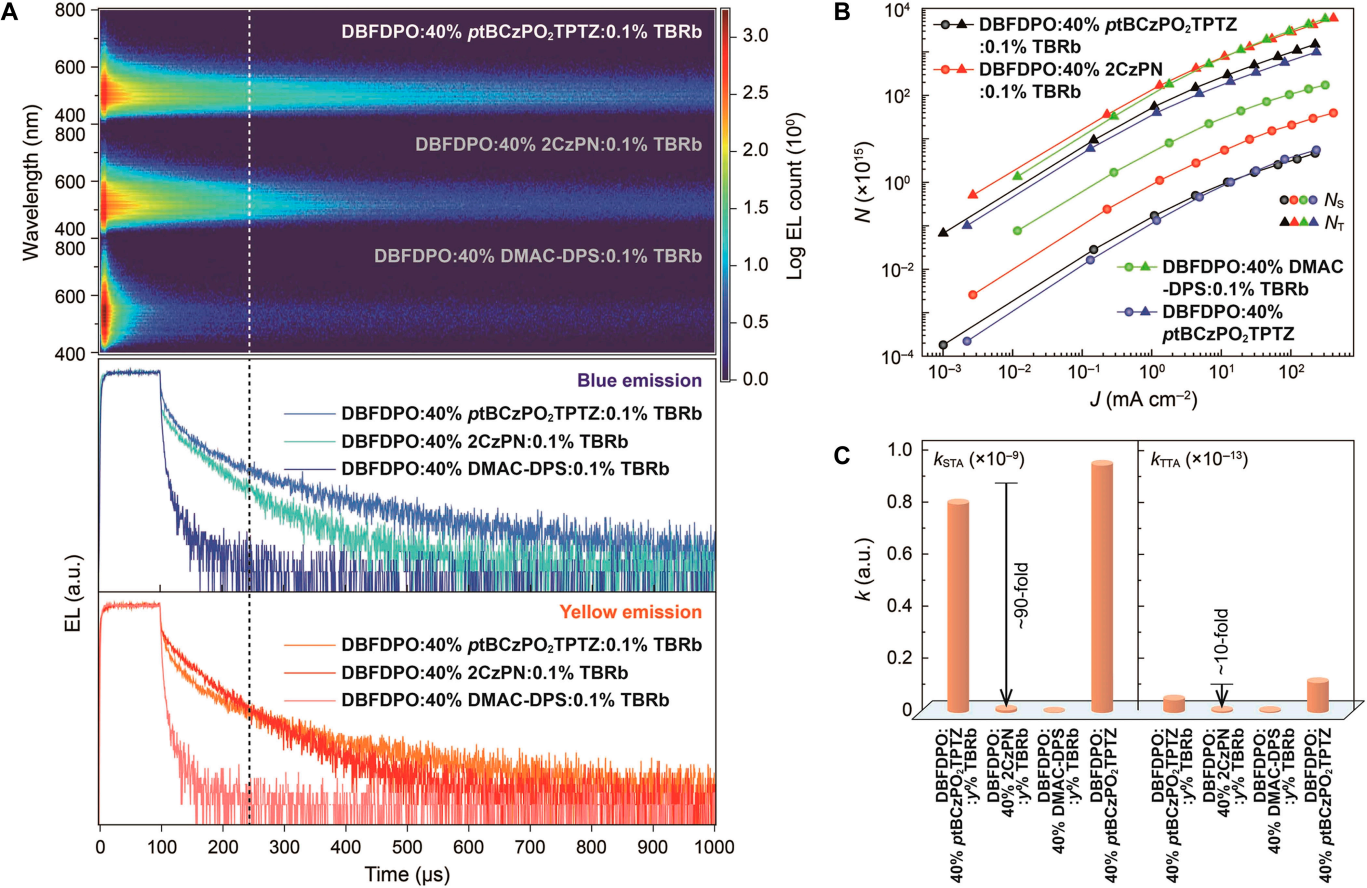
EL kinetics of white hyperfluorescence OLEDs. (A) Time-resolved EL emission spectra (top) and EL time decays at blue and yellow peak wavelengths (bottom) of devices with emissive layers of DBFDPO:40% blue TADF emitter:0.1% TBRb at 1,000 nits. (B) Relationship between singlet (*N*_S_, round symbols) and triplet (*N*_T_, triangle symbols) exciton densities and *J* in white OLEDs and blue diodes of DBFDPO:40% *p*tBCzPO_2_TPTZ. (C) Comparison on rate coefficients of singlet–triplet (STA, *k*_STA_) and triplet–triplet annihilations (TTA, *k*_TTA_) for the devices. a.u., arbitrary units.

Considering a singlet ratio of 25% and an outcoupling ratio of 20% to 30% for indium tin oxide (ITO) glass, an *η*_EQE_ of ~5% corresponds to 75% to 100% singlet utilization. Therefore, for a DMAC-DPS-based device with a maximum *η*_EQE_ of ~5%, DF was almost negligible. In contrast, *p*tBCzPO_2_TPTZ- and 2CzPN-based devices exhibited ~100% and 50% singlet and triplet utilization, respectively. Furthermore, EL TRES shows that the DF intensity of the DBFDPO:40% *p*tBCzPO_2_TPTZ:0.1% TBRb-based device was markedly higher than that of the 2CzPN-based analog (Fig. 4A). The blue DF EL lifetime of *p*tBCzPO_2_TPTZ-based devices was largely longer than that of the 2CzPN-based analog, manifesting the reduced triplet quenching on *p*tBCzPO_2_TPTZ. The situation of yellow DF EL lifetime was similar. It is noted that within 150 μs, the yellow intensity of the 2CzPN-based device was stronger than that of the *p*tBCzPO_2_TPTZ-based analog. However, after 150 μs, the yellow DF intensity of the former was, in turn, smaller than that of the latter. This variation is consistent with faster triplet DET and simultaneously stronger triplet quenching in the former.

Singlet (*N*_S_) and triplet (*N*_T_) exciton densities of DBFDPO:40% Blue TADF emitters:0.1% TBRb devices can be calculated as a function of current density (*J*)(*18*) (Fig. 4B):

$$\begin{array}{cc} \frac{dN_{S}}{\mathrm{dt}} & =\\ & -(k_{r}^{S}+k_{\mathrm{ISC}})N_{S}+k_{\text{RISC}}N_{T}\\ & -k_{\mathrm{STA}}N_{S}N_{T}+\alpha k_{\mathrm{TTA}}N_{T}^{2}+\frac{J}{4\mathrm{de}} \end{array}$$ (1)

$$\begin{array}{cc} \frac{dN_{T}}{\mathrm{dt}} & =\\ & -(k_{\mathrm{nr}}^{T}+k_{\text{RISC}})N_{T}+k_{\mathrm{ISC}}N_{S}\\ & -(1+\alpha )k_{\mathrm{TTA}}N_{T}^{2}+\frac{3J}{4\mathrm{de}} \end{array}$$ (2)

in which *k*_ISC_ and *k*_RISC_ are rate constants of ISC and RISC, and *d* and *e* are recombination zone thickness and electron charge, respectively. *k*_STA_ and *k*_TTA_ are rate constants of singlet–triplet and triplet–triplet annihilations, respectively. The *N*_S_ and *N*_T_ values were in the ranges of 10^11^ to 10^17^ and 10^14^ to 10^19^, respectively. It is shown that the *N*_S_ and *N*_T_ of DBFDPO:40% *p*tBCzPO_2_TPTZ:0.1% TBRb are almost the same as those of DBFDPO:40% *p*tBCzPO_2_TPTZ, manifesting the predominant roles of *p*tBCzPO_2_TPTZ in exciton conversion and utilization. Notably, *k*_STA_ and *k*_TTA_ values of *p*tBCzPO_2_TPTZ-based devices reaching the levels of 10^−10^ and 10^−15^ s^−1^ are comparable to those of FL OLEDs [45] but markedly higher than those of common TADF diodes [18] (Fig. 4C). *k*_STA_ and *k*_TTA_ are directly proportional to the oscillator strength of an exciton utilizer; therefore, it demonstrates that despite its charge transfer featured excited state, *p*tBCzPO_2_TPTZ (*η*_PL_ = 94%) is equal to locally excited TBRb (*η*_PL_ = 84%) in terms of exciton radiation, resulting in the balance of blue and yellow efficiencies for the state-of-the-art white OLEDs. In contrast to 2CzPN- and DMAC-DPS-based analogs, *p*tBCzPO_2_TPTZ significantly reduces *N*_S_ by about 30- and 400-fold, and *N*_T_ by 4- and 5-fold in its white OLEDs, respectively, and increases *k*_STA_ and *k*_TTA_ by more than 10- and 90-fold, respectively. Thus, blue TADF emitters are dominant in exciton kinetic processes of these hyperfluorescence white diodes. Undoubtedly, compared to 2CzPN and DMAC-DPS, the superiority of *p*tBCzPO_2_TPTZ in radiation facilitation and nonradiation suppression is the primary reason for the state-of-the-art performance of its white FL OLED.

## Discussion

We have demonstrated new record EL efficiencies of 30.7% for *η*_EQE_ and 120.2 lm W^−1^ for *η*_PE_ with a single-emissive layer white FL OLED, indicating the potential of pure organic OLEDs for large-scale daily lighting application. It is shown that 100% exciton utilization can be readily realized by hyperfluorescence white-emitting systems, when triplet DET and diffusion-induced quenching can be effectively suppressed. The fundamental solution is to reduce triplet concentration in the device, which requires TADF sensitizers to have ~100% *ϕ*_RISC_ and low enough $k_{\mathrm{nr}}^{T}$. Simultaneously, TADF sensitizers with a large steric hindrance are advantageous in reducing triplet diffusion to FL emitters. It is noted that FRET from eligible TADF materials, like *p*tBCzPO_2_TPTZ herein, to FL emitter can further improve RISC and singlet radiation and alleviate triplet nonradiation. These results provide new insights into optimizing exciton allocation and utilization in TADF-based systems. We obtained a power efficiency of 65.3 lm W^−1^ at 1,000 cd m^−2^, which can be doubled to over 130 lm W^−1^ by additional outcoupling enhancement, thereby surpassing the current benchmarks of daily lightings (100 lm W^−1^ for white LED and 70 lm W^−1^ for FL tube). For large-scale applications, the lifetime issue of blue TADF emitters should be solved in the future. Nevertheless, our results suggest that pure organic white OLEDs hold the promise of satisfying all demands for ideal lighting sources, including high efficiency, low cost, ecofriendliness, and so on.

## Materials and Methods

### Transient emission measurement

The films for measurement were prepared by vacuum evaporation. PL TRES were measured by an Edinburgh FLS 1000 fluorescence spectrophotometer using a time-correlated single photon counting method with a nanosecond and a microsecond pulsed light source for 100 ps to 10 s lifetime measurement, a synchronization photomultiplier for signal collection, and a multichannel scaling mode of the PCS900 fast counter PC plug-in card for data processing.

### Fabrication and characterization of OLEDs

The ITO substrate was cleaned with detergent and deionized water, dried in an oven at 120 °C for 4 h, treated with oxygen plasma for 3 min, and then transferred to a deposition chamber. Devices were fabricated by evaporating each layer onto the ITO substrate sequentially at a pressure below 1 × 10^−6^ Torr. A MoO_3_ layer was first deposited on the ITO substrate at 0.1 nm s^−1^. The deposition rates of organic layers were 0.1 to 0.3 nm s^−1^. Then, a 1-nm layer of LiF was deposited at 0.1 nm s^−1^. Finally, a 100-nm-thick layer of Al was deposited at 0.6 nm s^−1^ as the cathode. The devices were then transferred to a glovebox and encapsulated with hot melt glue. The emission areas of the devices were 0.09 cm^2^. EL spectra were measured with a PR655 spectra colorimeter. Current–density–voltage and brightness–voltage curves of the devices were measured using a Keithley 4200 source meter and a calibrated silicon photodiode. All the measurements were carried out in atmosphere.

## Data Availability

All other data are available from the authors upon reasonable request.

## Supplementary Materials

Supplementary Materials Experimental Details Fig. S1. Photoluminescence (PL) spectra and photoluminescence quantum yields (PLQY, *η*_PL_) of DBFDPO:*x*% *p*tBCzPO_2_TPTZ:0.1% TBRb films at different *x*. Fig. S2. Comparison on PL spectra of blue TADF emitter-based films before and after doping 0.1% TBRb. Bis{2-[di(phenyl)phosphino]-phenyl}ether oxide (DPEPO) and DBFDPO are used as host matrixes. Fig. S3. PL spectra and *η*_PL_ of DBFDPO:40% *p*tBCzPO_2_TPTZ:*y*% TBRb films at different *y*. Fig. S4. PL spectra and *η*_PL_ of *p*tBCzPO_2_TPTZ:*y*% TBRb films at different *y*. Fig. S5. TRES spectra of DBFDPO:40% 2CzPN:*y*% TBRb (above) and DBFDPO:40% DMAC-DPS:*y*% TBRb (below) films at different *y*. Fig. S6. TRES spectra of DBFDPO:*x*% *p*tBCzPO_2_TPTZ:*y*% TBRb films at different *x* and *y*. Fig. S7. TRES spectra of DPEPO:40% *p*tBCzPO_2_TPTZ:*y*% TBRb films at different *y*. Fig. S8. Prompt fluorescence (PF, insets) and delayed fluorescence (DF) time decay curves of (A) blue and (B) yellow components for blue TADF emitter-based films and the DBFDPO:5% TBRb film for comparison. Fig. S9. Prompt fluorescence (PF, insets) and delayed fluorescence (DF) time decay curves of (A) blue and (B) yellow components for DBFDPO:40% *p*tBCzPO_2_TPTZ:*y*% TBRb films at different *y*. Yellow DF lifetime variation is consistent with blue DF, but yellow PF lifetimes are slightly increased, due to the direct excitation and radiation of TBRb. Fig. S10. Prompt fluorescence (PF, insets) and delayed fluorescence (DF) time decay curves of (A) blue and (B) yellow components for DBFDPO:*x*% *p*tBCzPO_2_TPTZ:0.1% TBRb films at different *x*. Fig. S11. EL performance of DBFDPO:*x*% *p*tBCzPO_2_TPTZ:0.1% TBRb-based devices. (A) Current density (*J*)–voltage–luminance relationship and EL spectra at 1,000 nits (inset). (B) Efficiencies vs. luminance correlations. Fig. S12. EL performance of DBFDPO:*x*% 2CzPN:0.1% TBRb-based devices. (A) Current density (*J*)–voltage–luminance relationship and EL spectra at 1,000 nits (inset). (B) Efficiencies vs. luminance correlations. Fig. S13. EL performance of DBFDPO:*x*% DMAC-DPS:0.1% TBRb-based devices. (A) Current density (*J*)–voltage–luminance relationship and EL spectra at 1,000 nits (inset). (B) Efficiencies vs. luminance correlations. Fig. S14. EL performance of DBFDPO:40% *p*tBCzPO_2_TPTZ:*y*% TBRb-based devices. (A) Current density (*J*)–voltage–luminance relationship and EL spectra at 1,000 nits (inset). (B) Efficiencies vs. luminance correlations. Fig. S15. EL performance of *p*tBCzPO_2_TPTZ:*y*% TBRb-based devices. (A) Current density (*J*)–voltage–luminance relationship and EL spectra at 1,000 nits (inset). (B) Efficiencies vs. luminance correlations. Fig. S16. EL performance of DPEPO:*x*% *p*tBCzPO_2_TPTZ:0.1% TBRb-based devices. (A) Current density (*J*)–voltage–luminance relationship and EL spectra at 1,000 nits (inset). (B) Efficiencies vs. luminance correlations. Fig. S17. Chromatic properties of DBFDPO:40% *p*tBCzPO_2_TPTZ:*y*% TBRb-based white OLEDs. Fig. S18. Dependence of the maximum EQE values on doping concentrations of DBFDPO:*x*% *p*tBCzPO_2_TPTZ:*y*% TBRb-based devices. Table S1. Photophysical properties of *p*tBCzPO_2_TPTZ-, 2CzPN-, and DMAC-DPS-based films. Table S2. Device performance of DBFDPO:*x*% blue TADF emitter:*y*% TBRb *p*tBCzPO_2_TPTZ- and 2CzPN-based white OLEDs. Table S3. EL performance of representative TADF-involved white OLEDs.
