# Supplementary material for: White Fluorescent Organic Light-Emitting Diodes with 100% Power Conversion
Source: Research (Wash D C). 2022 Dec 19;2022:0009. doi: 10.34133/research.0009 (PMC11407583; doi:10.34133/research.0009)
Supplement: Supplementary Materials — Experimental Details Fig. S1. Photoluminescence (PL) spectra and photoluminescence quantum yields (PLQY, ηPL) of DBFDPO:x% ptBCzPO2TPTZ:0.1% TBRb films at different x. Fig. S2. Comparison on PL spectra of blue TADF emitter-based films before and after doping 0.1% TBRb. Bis{2-[di(phenyl)phosphino]-phenyl}ether oxide (DPEPO) and DBFDPO are used as host matrixes. Fig. S3. PL spectra and ηPL of DBFDPO:40% ptBCzPO2TPTZ:y% TBRb films at different y. Fig. S4. PL spectra and ηPL of ptBCzPO2TPTZ:y% TBRb films at different y. Fig. S5. TRES spectra of DBFDPO:40% 2CzPN:y% TBRb (above) and DBFDPO:40% DMAC-DPS:y% TBRb (below) films at different y. Fig. S6. TRES spectra of DBFDPO:x% ptBCzPO2TPTZ:y% TBRb films at different x and y. Fig. S7. TRES spectra of DPEPO:40% ptBCzPO2TPTZ:y% TBRb films at different y. Fig. S8. Prompt fluorescence (PF, insets) and delayed fluorescence (DF) time decay curves of (A) blue and (B) yellow components for blue TADF emitter-based films and the DBFDPO:5% TBRb film for comparison. Fig. S9. Prompt fluorescence (PF, insets) and delayed fluorescence (DF) time decay curves of (A) blue and (B) yellow components for DBFDPO:40% ptBCzPO2TPTZ:y% TBRb films at different y. Yellow DF lifetime variation is consistent with blue DF, but yellow PF lifetimes are slightly increased, due to the direct excitation and radiation of TBRb. Fig. S10. Prompt fluorescence (PF, insets) and delayed fluorescence (DF) time decay curves of (A) blue and (B) yellow components for DBFDPO:x% ptBCzPO2TPTZ:0.1% TBRb films at different x. Fig. S11. EL performance of DBFDPO:x% ptBCzPO2TPTZ:0.1% TBRb-based devices. (A) Current density (J)–voltage–luminance relationship and EL spectra at 1,000 nits (inset). (B) Efficiencies vs. luminance correlations. Fig. S12. EL performance of DBFDPO:x% 2CzPN:0.1% TBRb-based devices. (A) Current density (J)–voltage–luminance relationship and EL spectra at 1,000 nits (inset). (B) Efficiencies vs. luminance correlations. Fig. S13. EL performance of DBFDPO:x% [file research.0009.f1.docx]

Supplementary Materials

### White Fluorescent Organic Light-Emitting Diodes with 100% Power Conversion

Dongxue Ding^†^, Zicheng Wang^†^, Chunbo Duan, Chunmiao Han, Jing Zhang, Shuo Chen, Ying Wei*, Hui Xu*

^1^Key Laboratory of Functional Inorganic Material Chemistry (Ministry of Education) & School of Chemistry and Material Science, Heilongjiang University, 74 Xuefu Road, Harbin 150080, P. R. China.

*Corresponding authors.

E-mail: YW: [ywei@hlju.edu.cn](mailto:ywei@hlju.edu.cn); HX: [hxu@hlju.edu.cn](mailto:hxu@hlju.edu.cn).

Materials and Methods

Materials

*p*tBCzPO_2_TPTZ and *p*TPOTZ were synthesized according to our previous reports(*1, 2*). Other materials used for film preparation and device fabrication were purchased from Xi’An *p*-OLED technology company, and used after sublimation.

Methods

*Electrochemical analysis:* Cyclic voltammetry (CV) studies were conducted using an Eco Chemie B.V. AUTOLAB potentiostat in a typical three-electrode cell with a glassy carbon working electrode, a platinum wire counter electrode, and a silver/silver chloride reference electrode. All electrochemical experiments were carried out under a nitrogen atmosphere at room temperature in dichloromethane and tetrahydrofuran.

*Optical Measurement:* The films for measurement were prepared by vacuum evaporation. Absorption and PL emission spectra of the target compound were measured using a Shimadzu UV-3150 spectrophotometer and a Shimadzu RF-5301PC spectrophotometer, respectively. Transient emission spectra were measured by an Edinburgh FLS 1000 fluorescence spectrophotometer using a time-correlated single photon counting method with a nanosecond and a microsecond pulsed light sources for 100 ps-10 s lifetime measurement, synchronization photomultiplier for signal collection, and multi-channel scaling mode of the PCS900 fast counter PC plug-in card for data processing. Photoluminescent quantum yields (PLQY, *η*_PL_) of these films were measured through a Labsphere 1-M-2 integrating sphere (*ϕ* = 6 in.) coated with Benflect material with efficient light reflection in a wide range of 200−1600 nm, which was integrated with an FLS 1000 fluorescence spectrophotometer. The absolute *η*_PL_ of the sample was determined by performing two spectral (emission) scans, with the emission monochromator scanning over the Rayleigh scattered light from the sample and from a blank substrate. The first spectrum recorded the scattered light and the emission of the sample, and the second spectrum recorded the scattered light of the Benflect coating. Integration and subtraction of the scattered light parts of these two spectra provided the photon number absorbed by the samples (*N*a), while integration of the emission of the samples was done to calculate the emissive photon number (*N*e). The absolute *η*_PL_ can then be estimated according to the equation *η*_PL_ = *N*e/*N*a. Spectral correction (emission arm) was applied to the raw data after background subtraction, and from these spectrally corrected curves, the quantum yield was calculated using aF900 software wizard.

EL time-resolved emission spectra were recorded with the Edinburgh FLS1000 fluorescence spectrophotometer, based on TCSPC method, which was equipped with a Tektronix AFG3022C function generator for EL measurement. The devices were fabricated in a vacuum chamber to get rid of air influences.


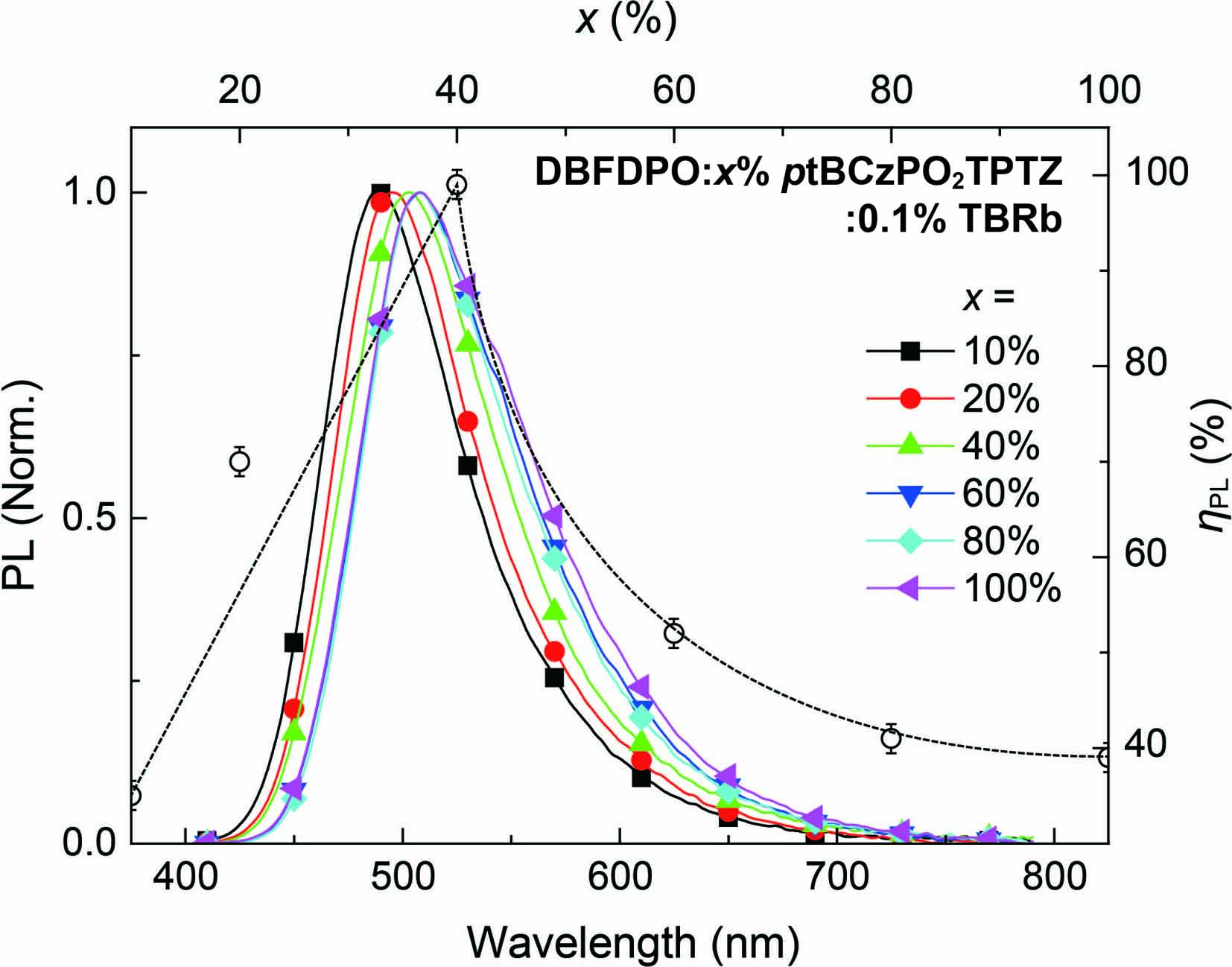


Fig. S1.

Photoluminescence (PL) spectra and photoluminescence quantum yields (PLQY, *η*_PL_) of DBFDPO:*x*% *p*tBCzPO_2_TPTZ:0.1% TBRb films at different *x*. Increasing *x* shortens distances between *p*tBCzPO_2_TPTZ and TBRb, therefore facilitates energy transfer. The enhanced yellow emission actually reflects the highly efficient blue-to-yellow energy transfer. When *x*% beyond 40%, the collision-induced triplet quenching and triplet leakage to nonradiative triplet state of TBRb become considerable and directly proportional to *x*%, which counteracts the increased triplet concentrations. However, since PL of TBRb originates from its singlet state and FRET from singlet state of *p*tBCzPO_2_TPTZ, the relatively stable singlet states lead to the small influence of *x*% on PL of TBRb.


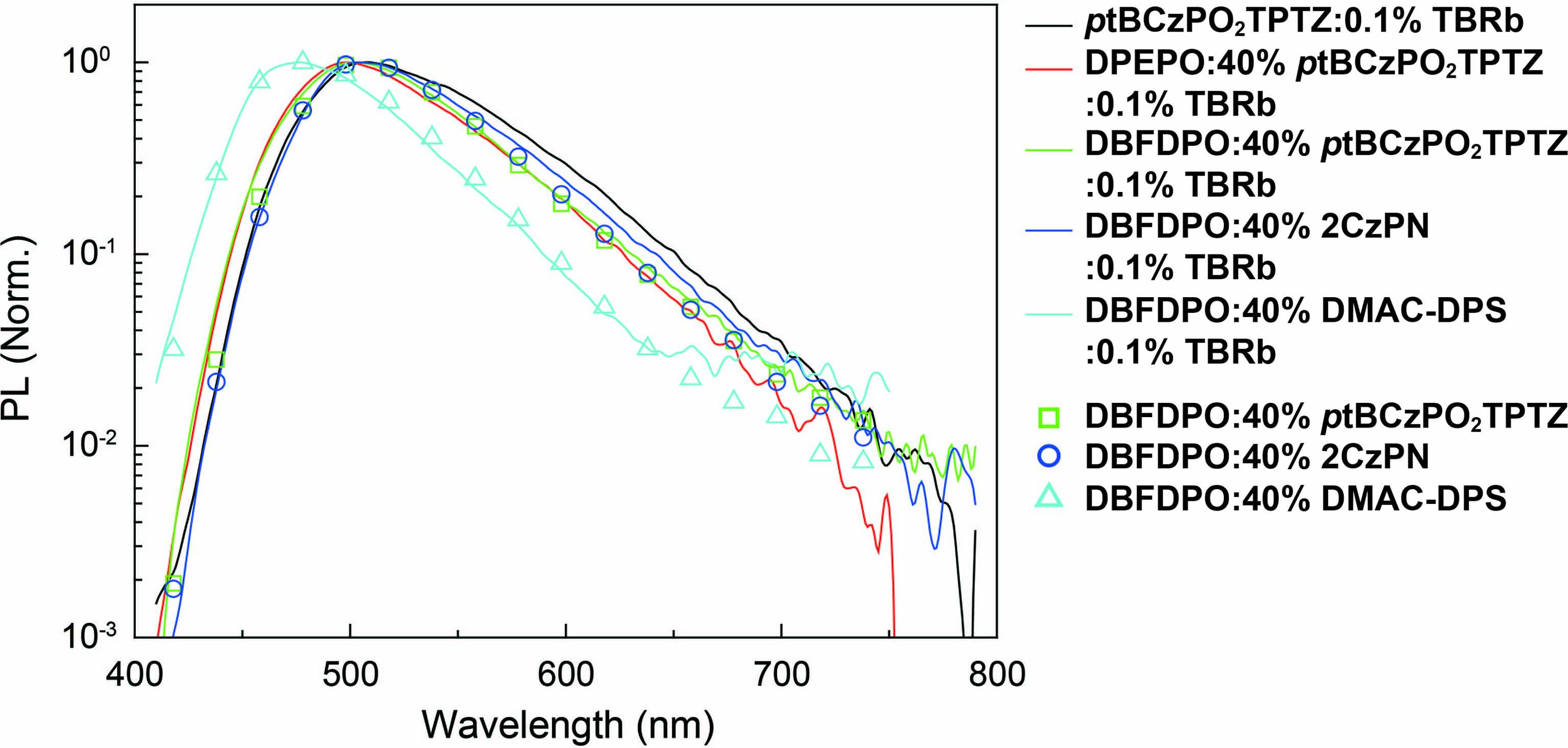


Fig. S2.

Comparison on PL spectra of blue TADF emitters based films before and after doping 0.1% TBRb. Bis{2-[di(phenyl)phosphino]-phenyl}ether oxide (DPEPO) and DBFDPO are used as host matrixes.


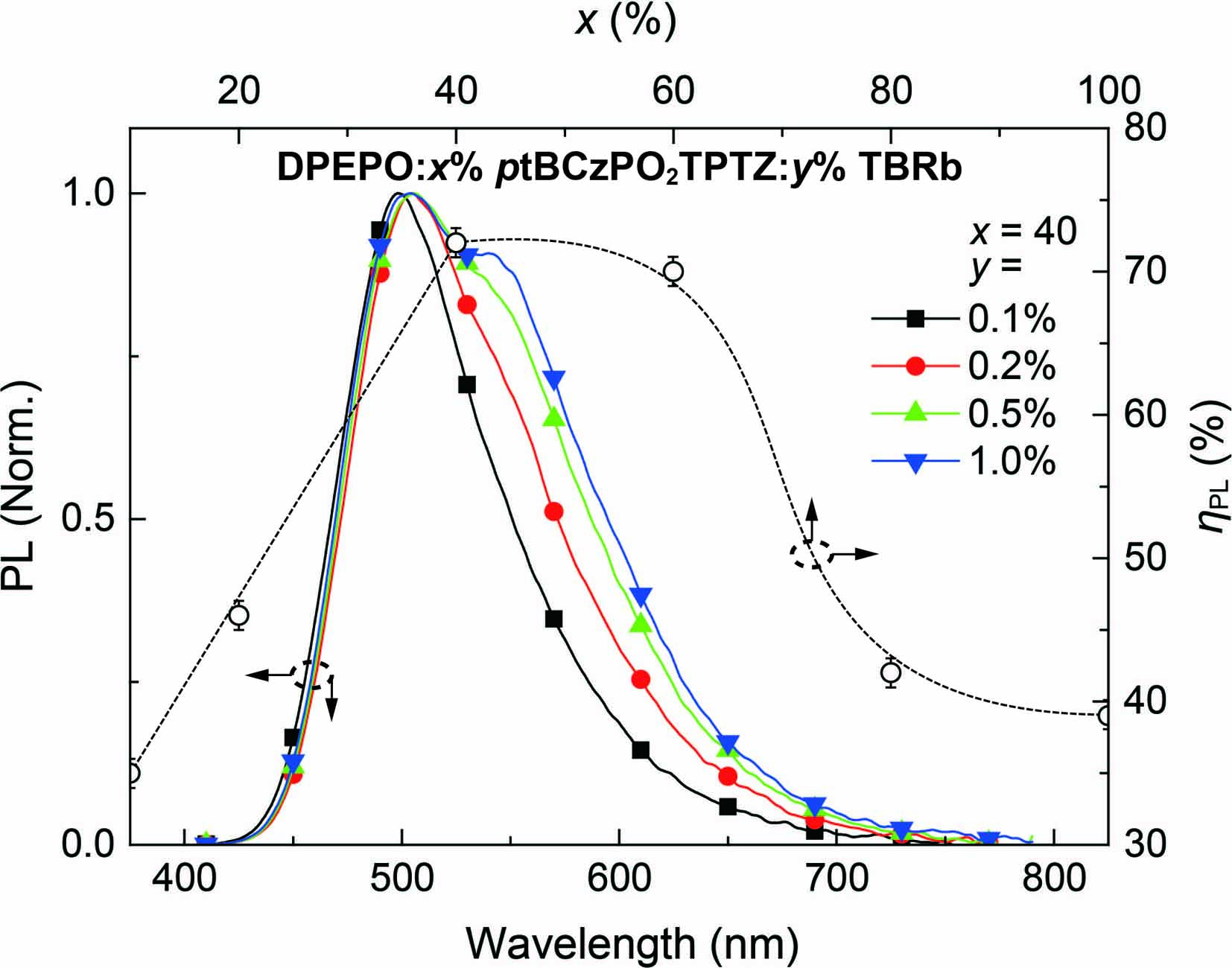


Fig. S3.

PL spectra and *η*_PL_ of DBFDPO:40% *p*tBCzPO_2_TPTZ:*y*% TBRb films at different *y*.


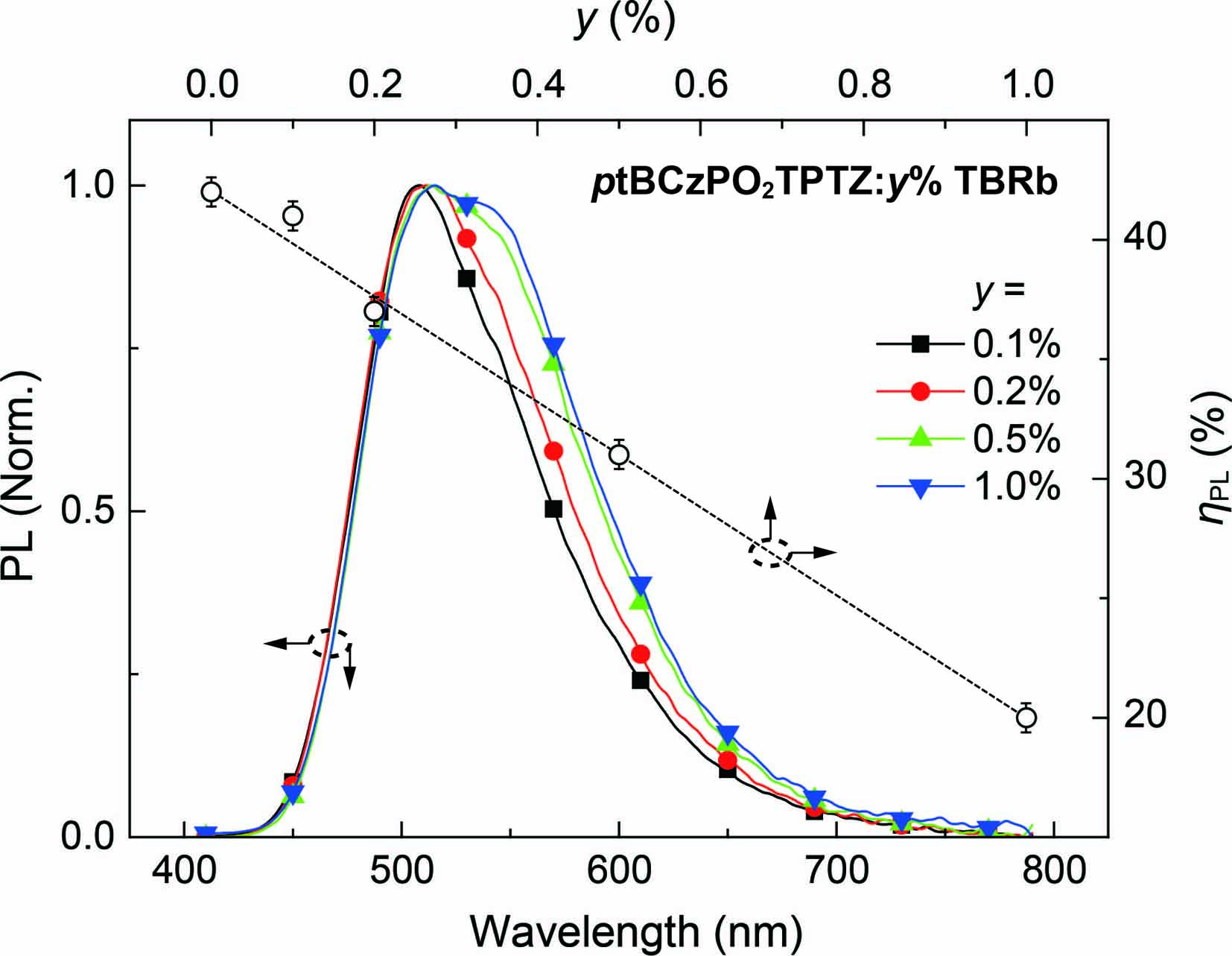


Fig. S4.

PL spectra and *η*_PL_ of *p*tBCzPO_2_TPTZ:*y*% TBRb films at different *y*.


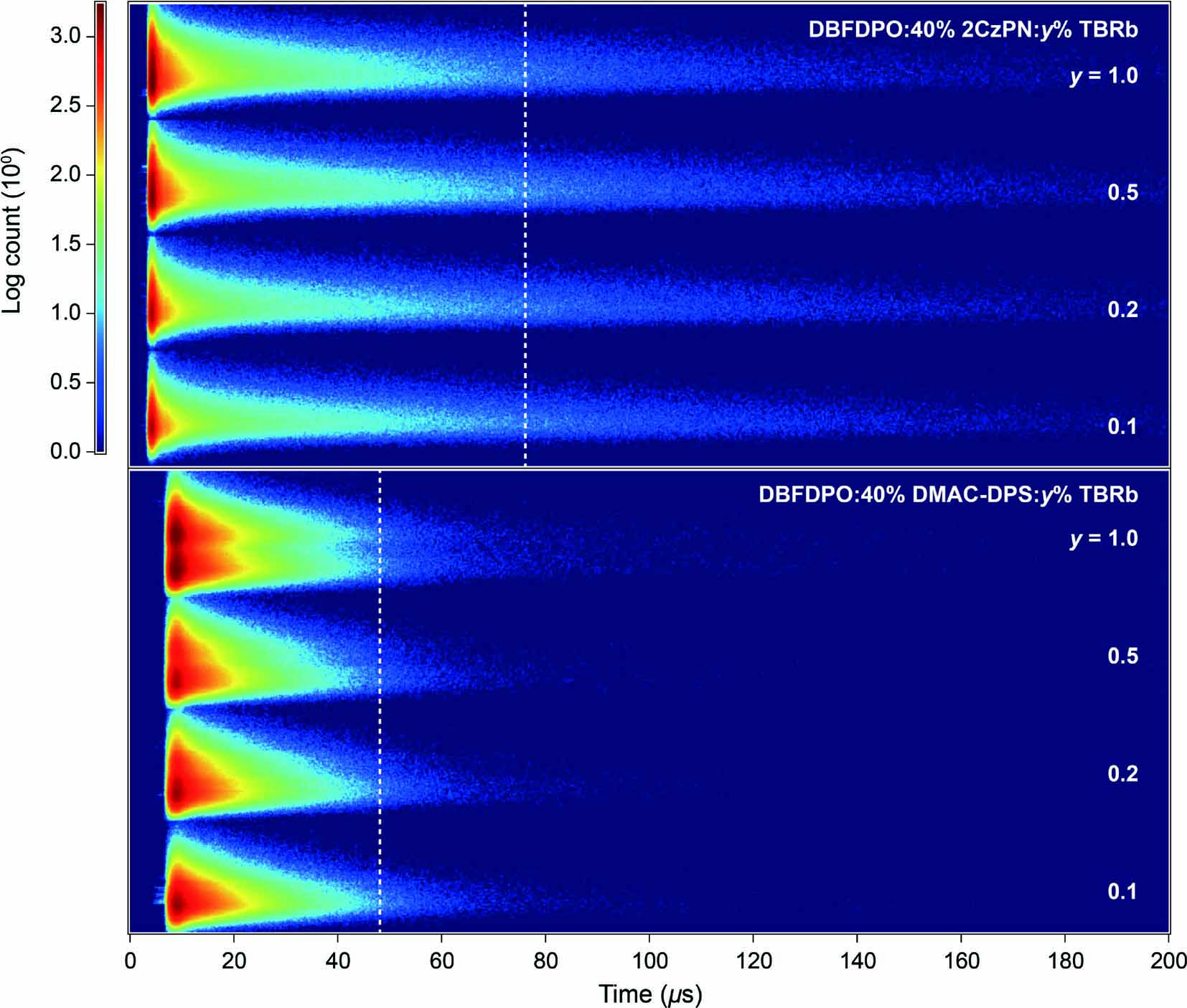


Fig. S5.

TRES spectra of DBFDPO:40% 2CzPN:*y*% TBRb (above) and DBFDPO:40% DMAC-DPS:*y*% TBRb (below) films at different *y*.


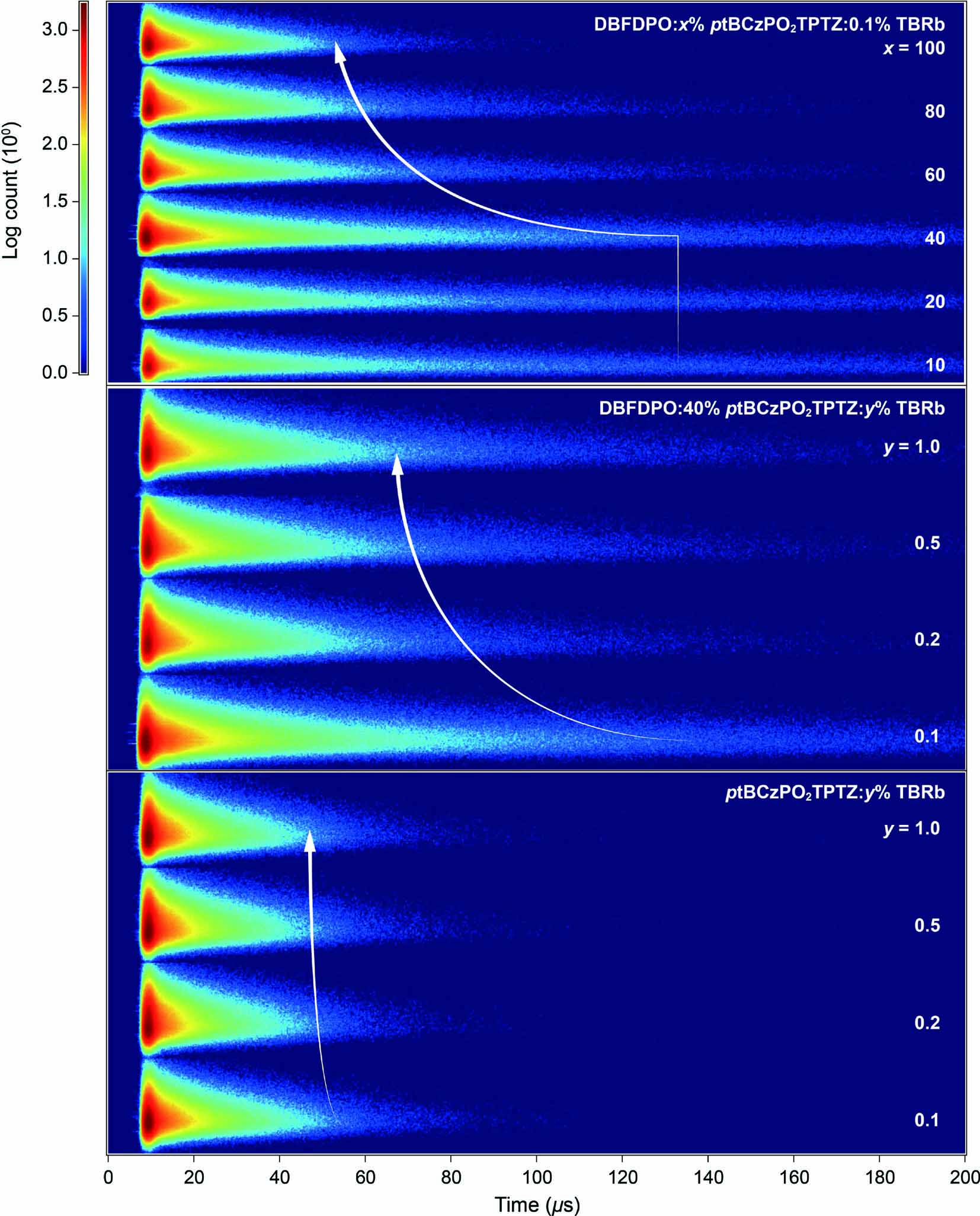


Fig. S6.

TRES spectra of DBFDPO:*x*% *p*tBCzPO_2_TPTZ:*y*% TBRb films at different *x* and *y*.


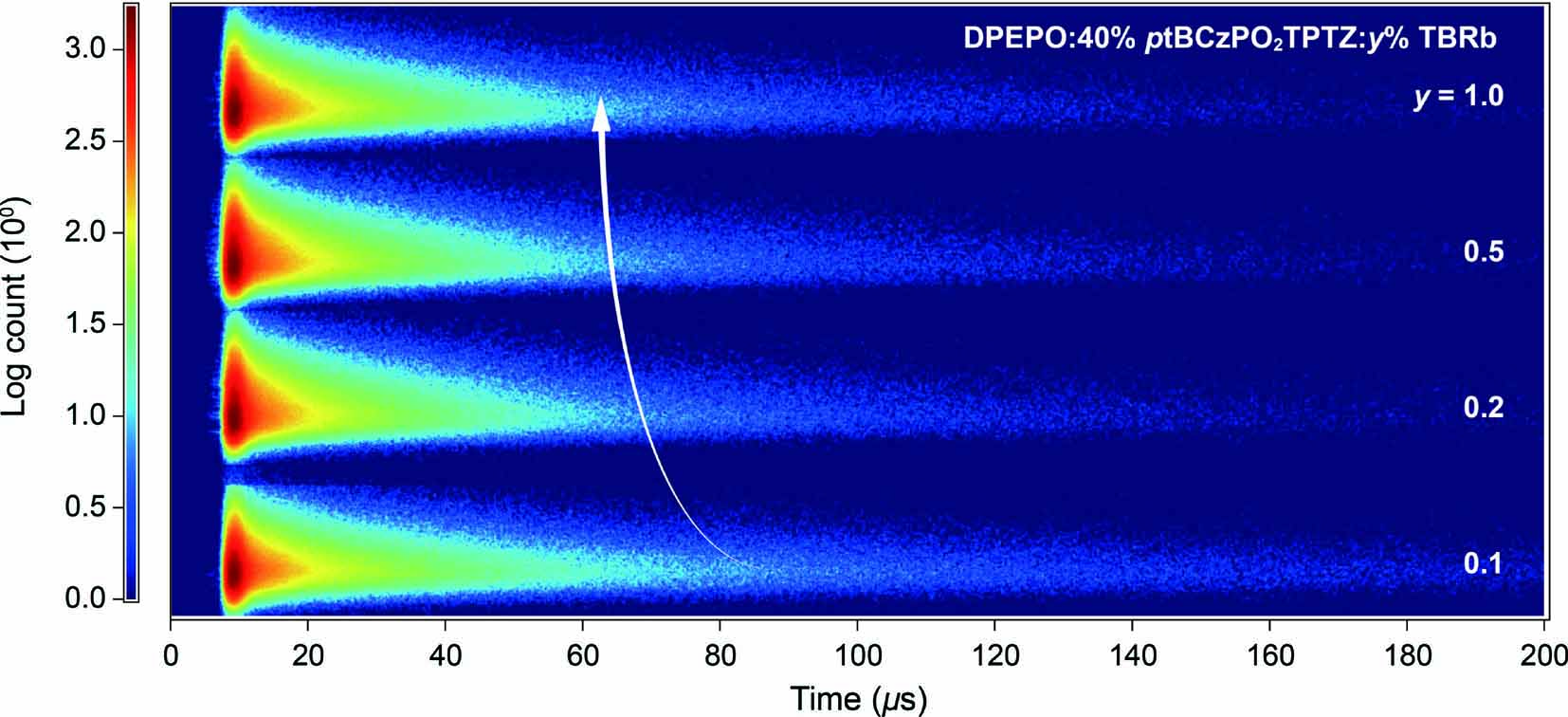


Fig. S7.

TRES spectra of DPEPO:40% *p*tBCzPO_2_TPTZ:*y*% TBRb films at different *y*.


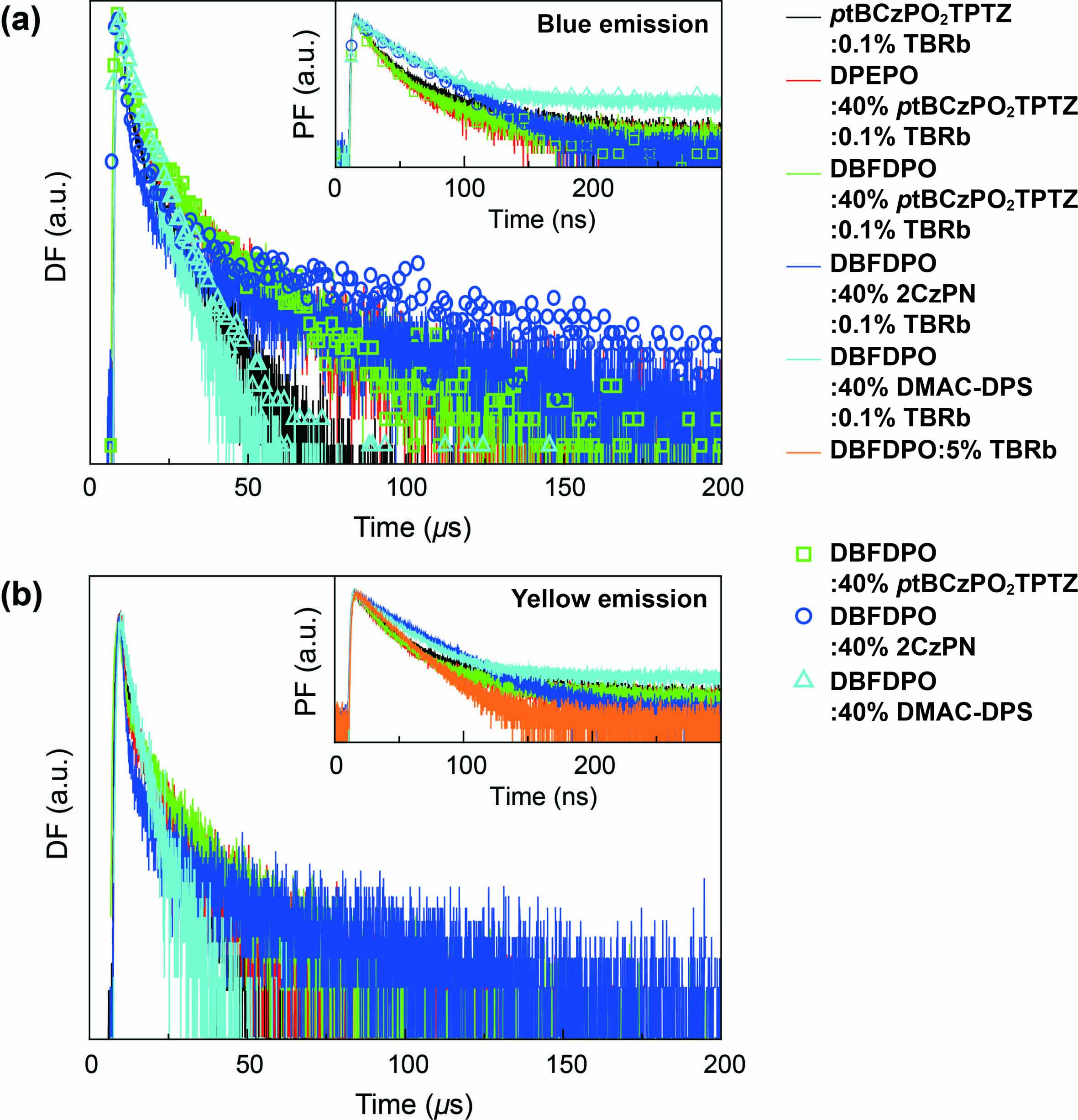


Fig. S8.

Prompt (PF, insets) and delayed fluorescence (DF) time decay curves of (**a**) blue and (**b**) yellow components for blue TADF emitters based films and DBFDPO:5% TBRb film for comparison.


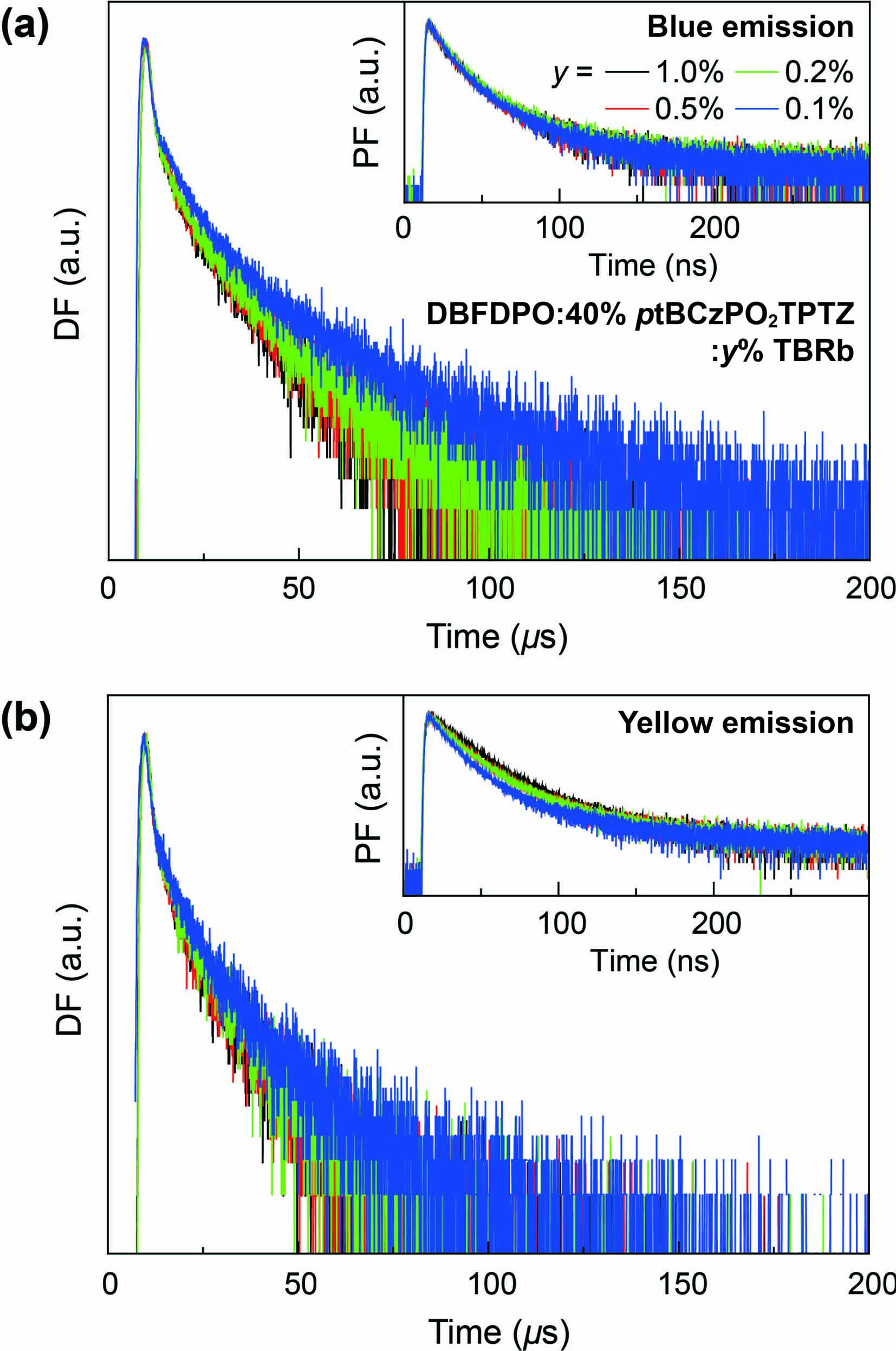


Fig. S9.

Prompt (PF, insets) and delayed fluorescence (DF) time decay curves of (**a**) blue and (**b**) yellow components for DBFDPO:40% *p*tBCzPO_2_TPTZ:*y*% TBRb films at different *y*. Yellow DF lifetime variation is consistent to blue DF, but yellow PF lifetimes are slightly increased, due to the direct excitation and radiation of TBRb.


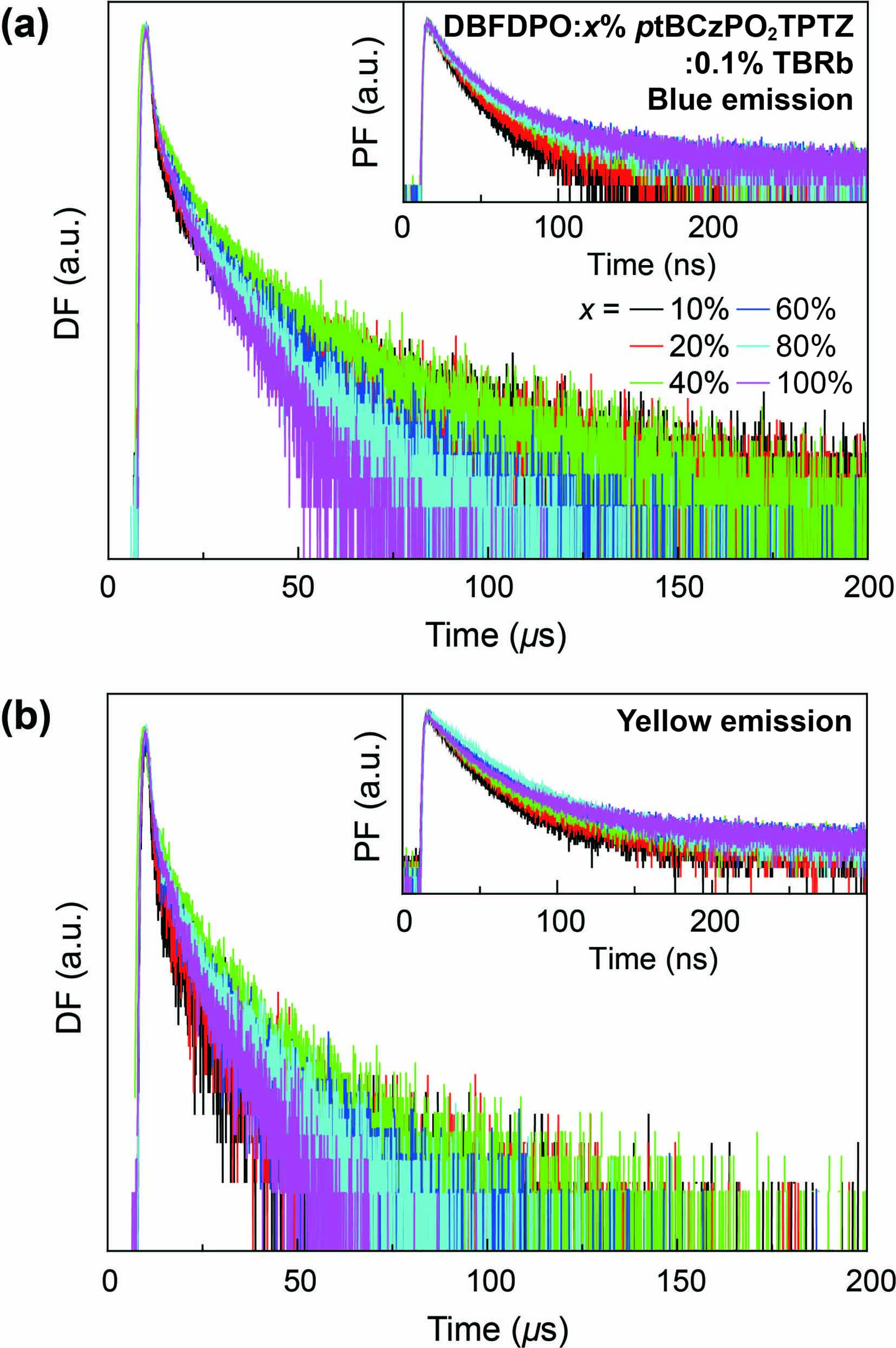


Fig. S10.

Prompt (PF, insets) and delayed fluorescence (DF) time decay curves of (**a**) blue and (**b**) yellow components for DBFDPO:*x*% *p*tBCzPO_2_TPTZ:0.1% TBRb films at different *x*.


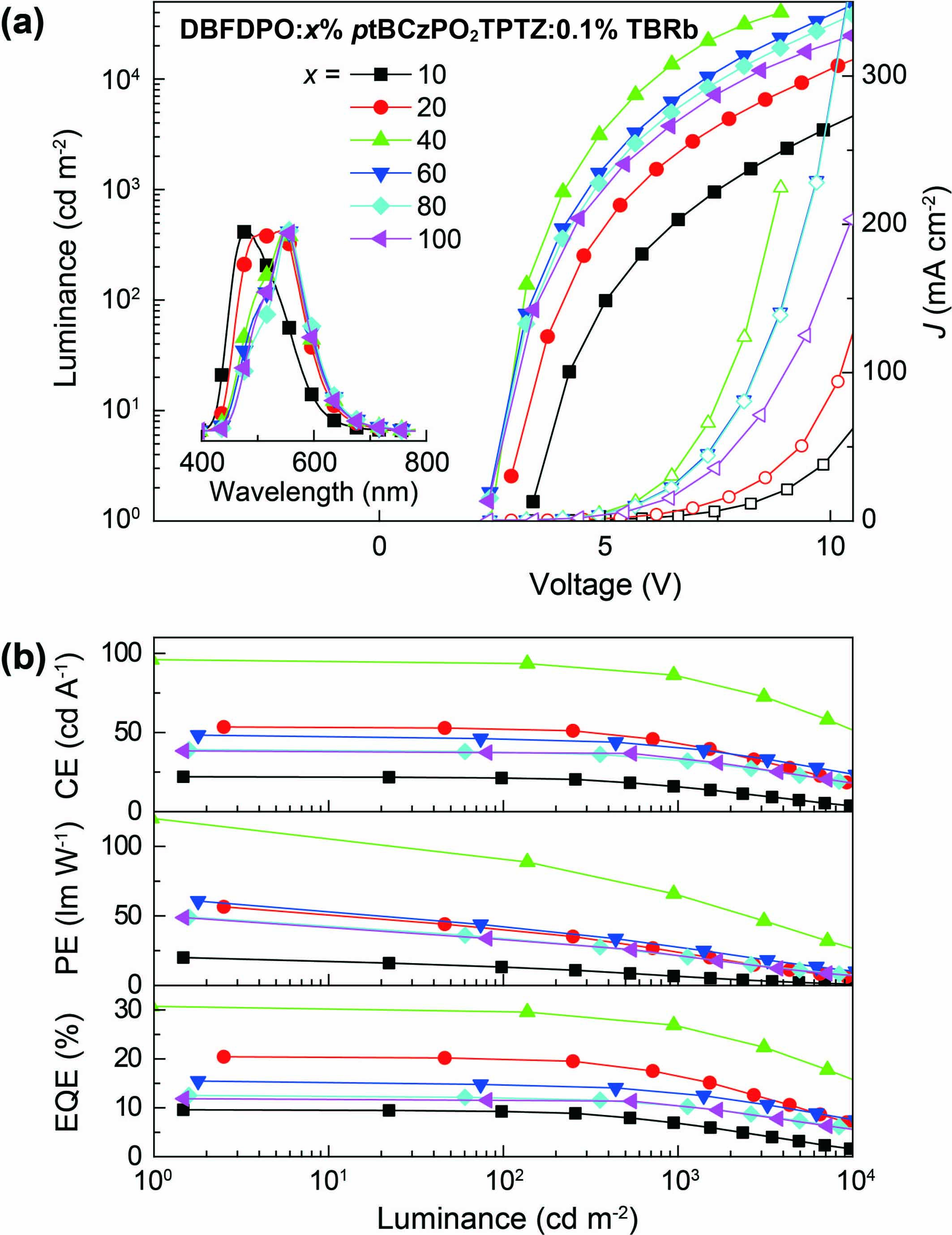


Fig. S11.

EL performance of DBFDPO:*x*% *p*tBCzPO_2_TPTZ:0.1% TBRb based devices. (**a**) Current density (*J*)-voltage-luminance relationship and EL spectra at 1000 nits (inset); (**b**) Efficiencies *vs.* luminance correlations.


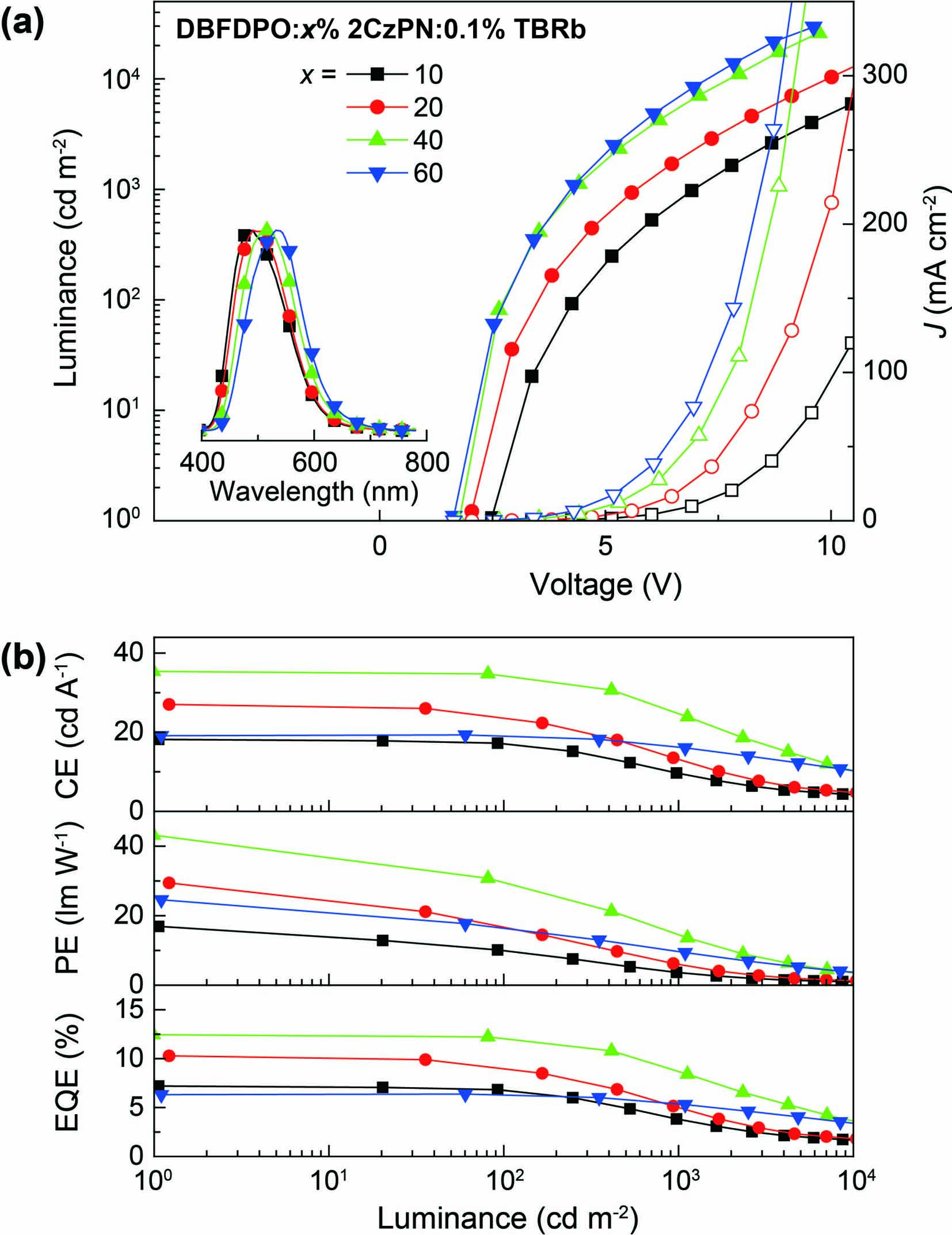


Fig. S12.

EL performance of DBFDPO:*x*% 2CzPN:0.1% TBRb based devices. (**a**) Current density (*J*)-voltage-luminance relationship and EL spectra at 1000 nits (inset); (**b**) Efficiencies *vs.* luminance correlations.


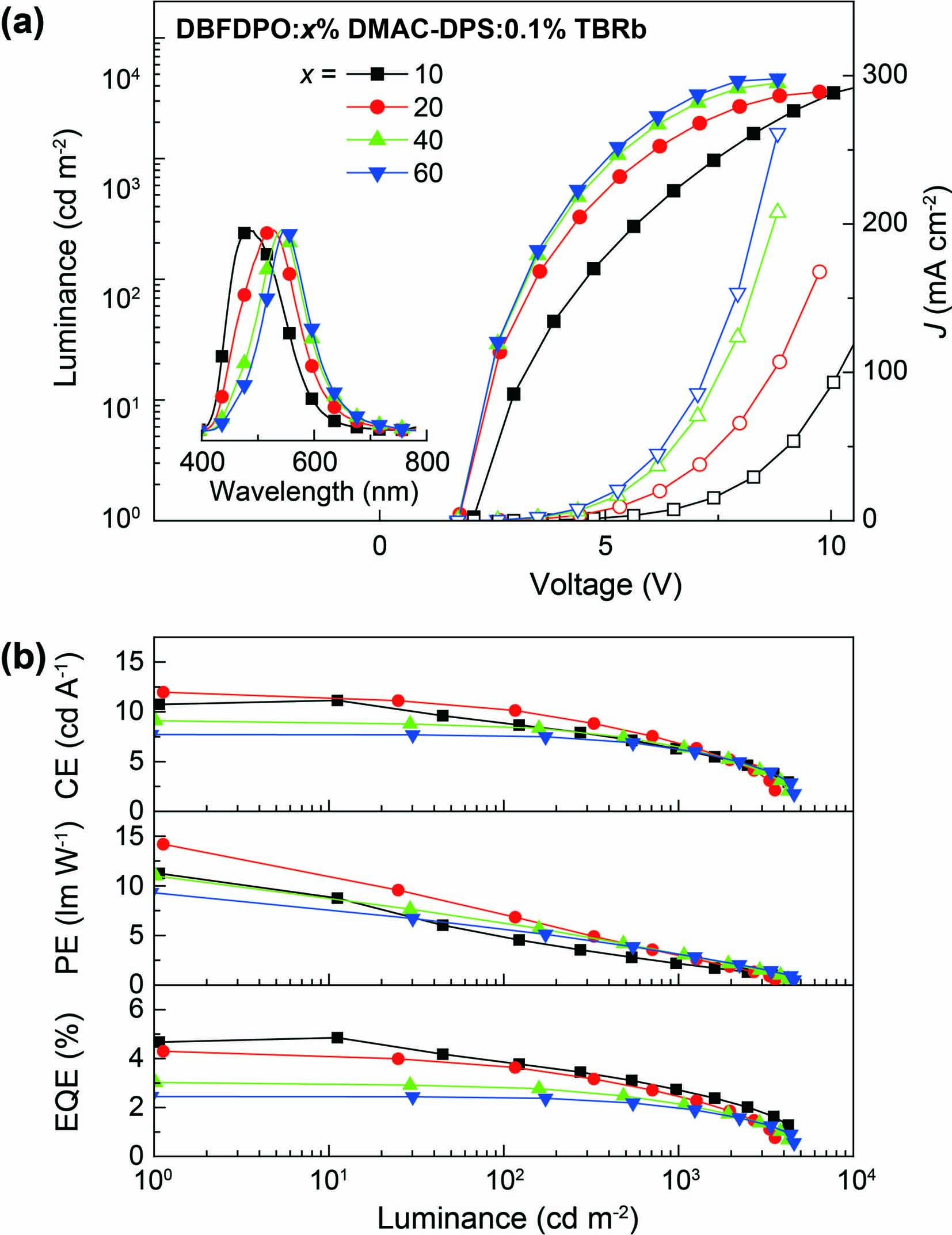


Fig. S13.

EL performance of DBFDPO:*x*% DMAC-DPS:0.1% TBRb based devices. (**a**) Current density (*J*)-voltage-luminance relationship and EL spectra at 1000 nits (inset); (**b**) Efficiencies *vs.* luminance correlations.


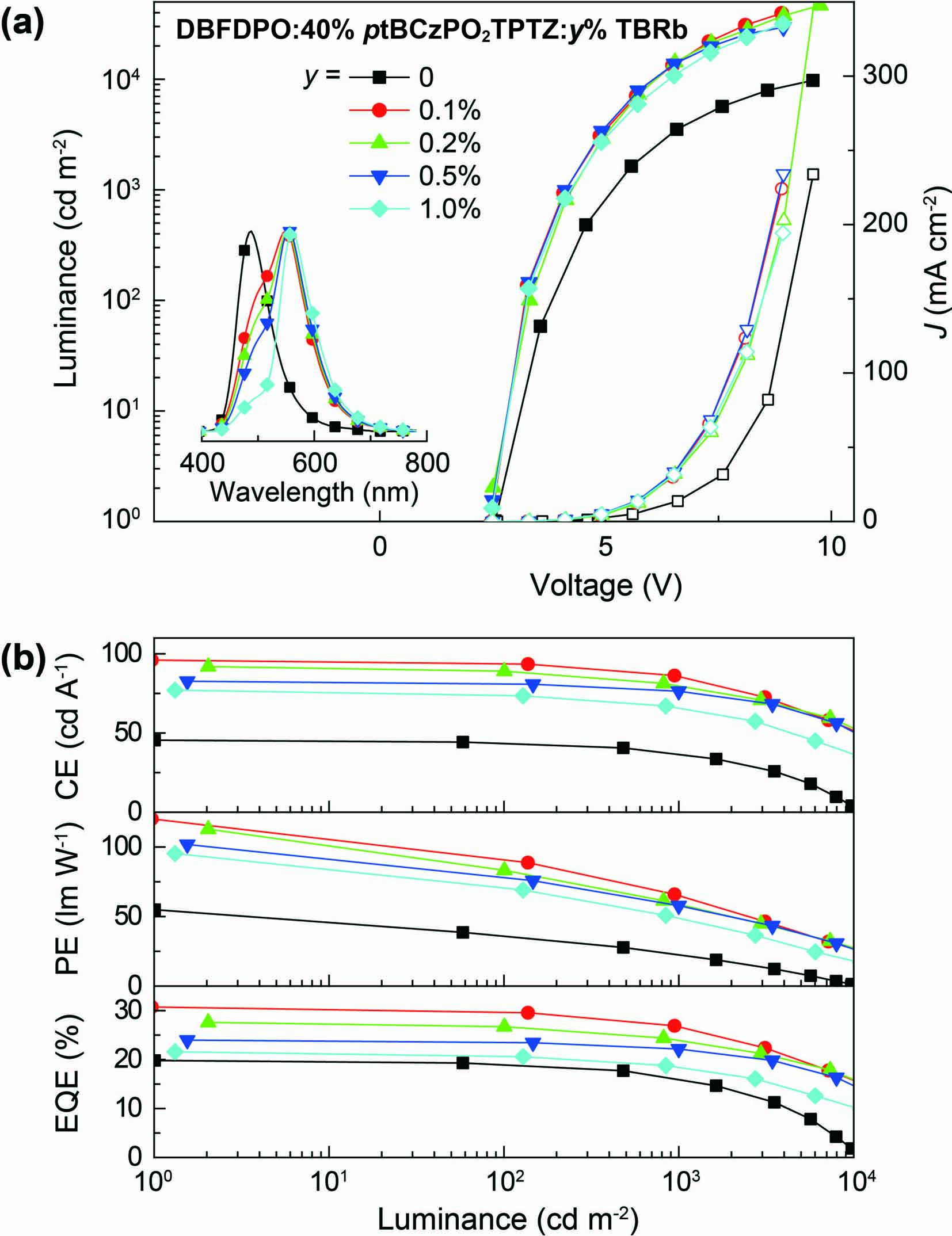


Fig. S14.

EL performance of DBFDPO:40% *p*tBCzPO_2_TPTZ:*y*% TBRb based devices. (**a**) Current density (*J*)-voltage-luminance relationship and EL spectra at 1000 nits (inset); (**b**) Efficiencies *vs.* luminance correlations.


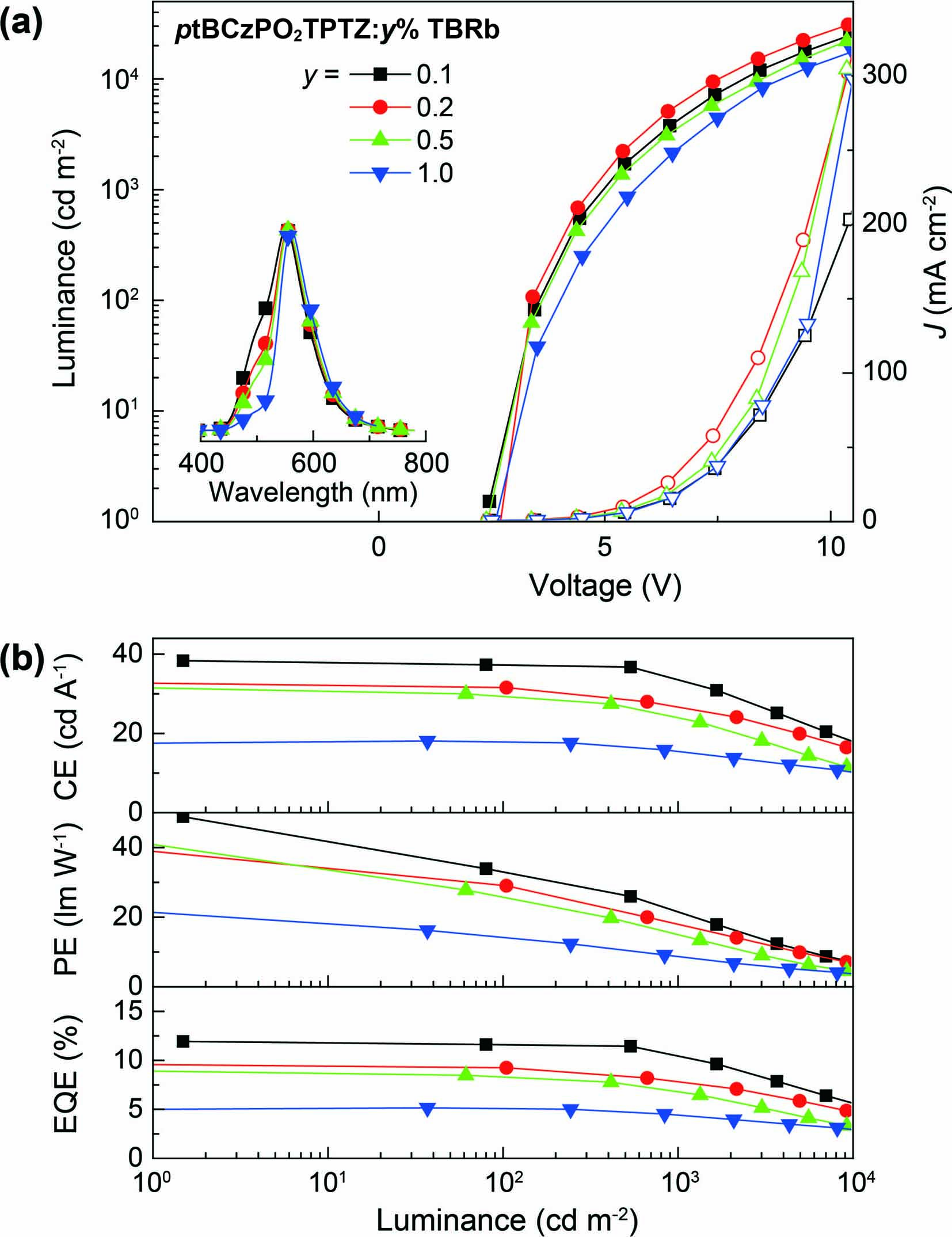


Fig. S15.

EL performance of *p*tBCzPO_2_TPTZ:*y*% TBRb based devices. (**a**) Current density (*J*)-voltage-luminance relationship and EL spectra at 1000 nits (inset); (**b**) Efficiencies *vs.* luminance correlations.


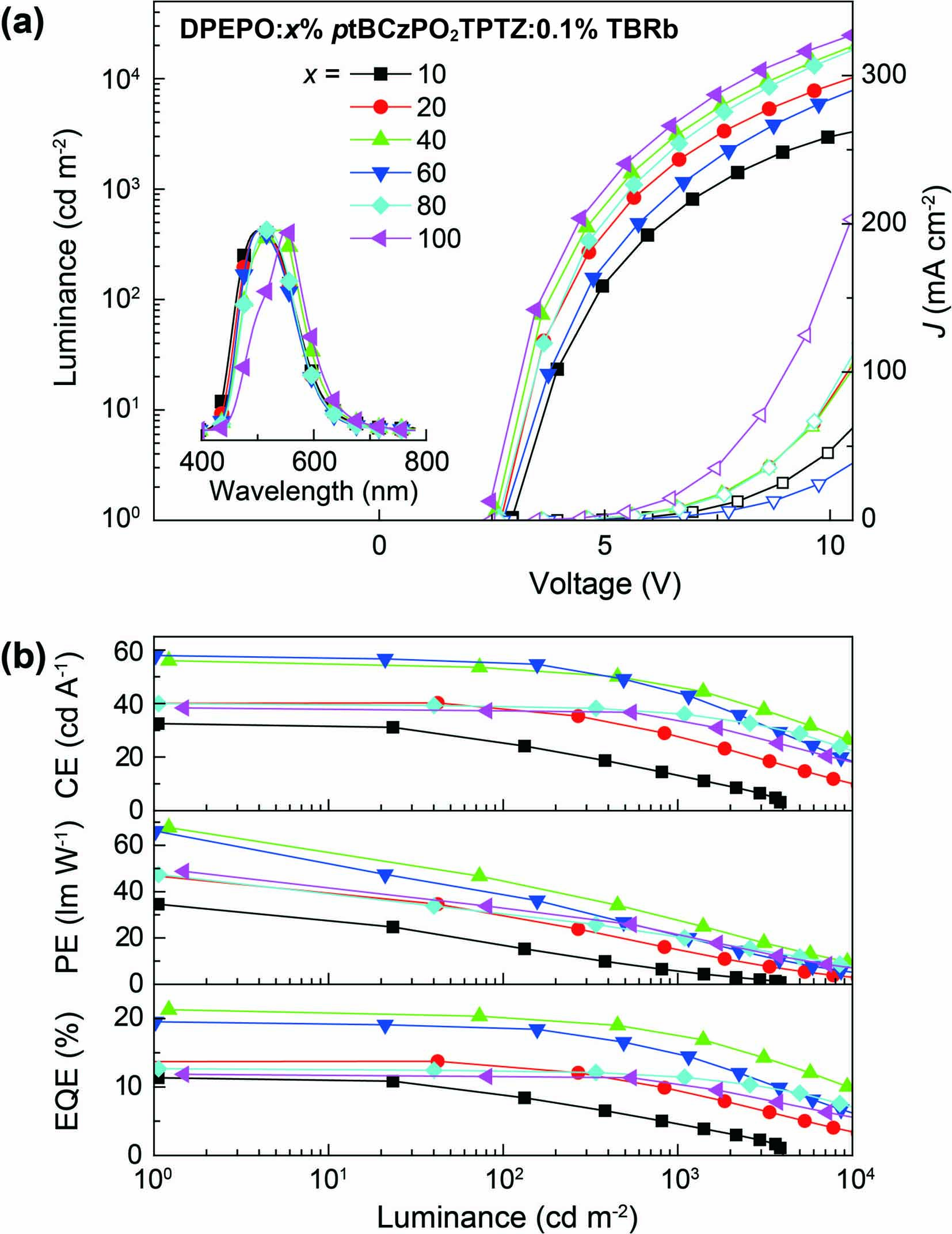


Fig. S16.

EL performance of DPEPO:*x*% *p*tBCzPO_2_TPTZ:0.1% TBRb based devices. (**a**) Current density (*J*)-voltage-luminance relationship and EL spectra at 1000 nits (inset); (**b**) Efficiencies *vs.* luminance correlations.


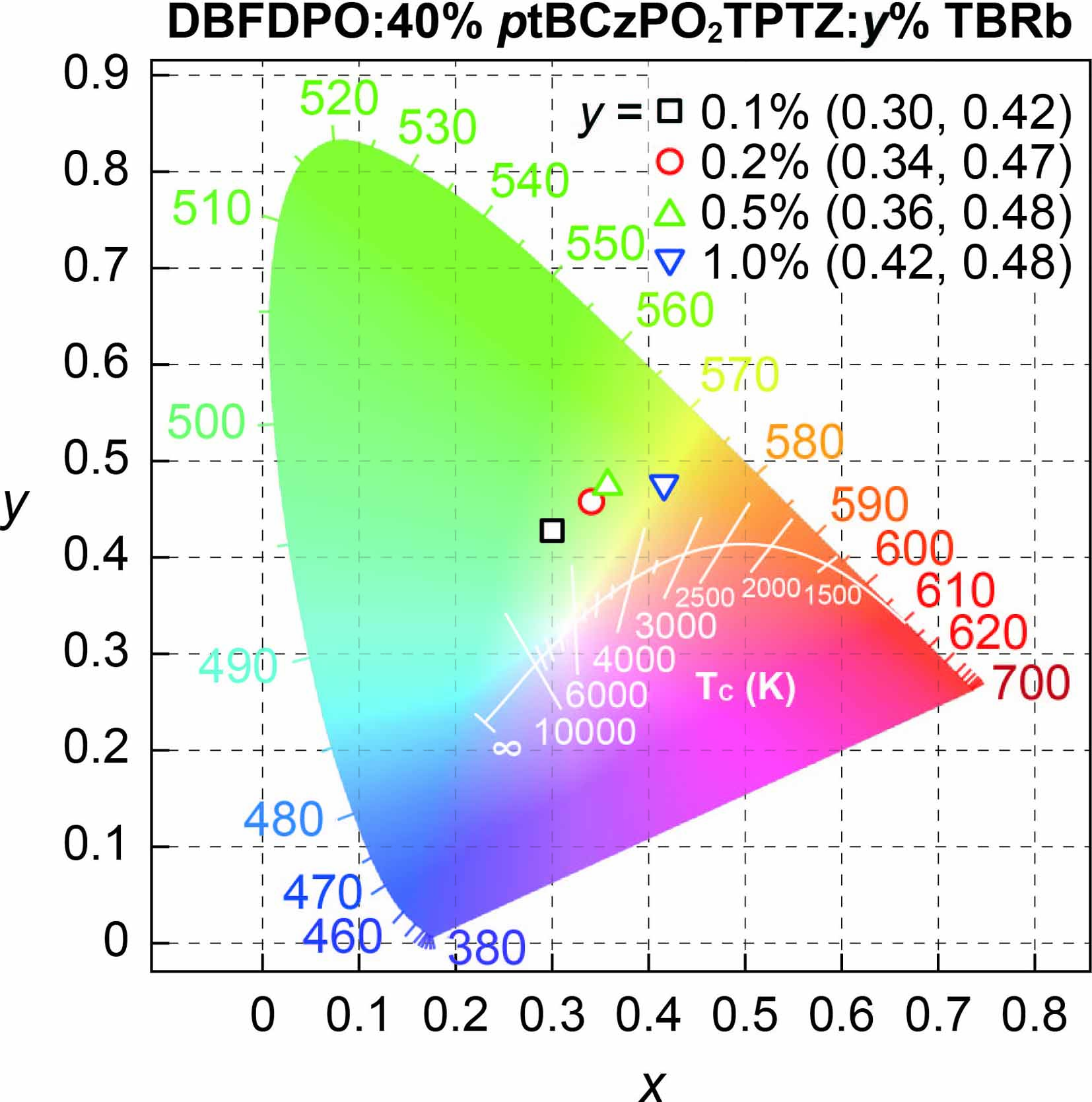


Fig. S17.

Chromatic properties of DBFDPO:40% *p*tBCzPO_2_TPTZ:*y*% TBRb based white OLEDs.


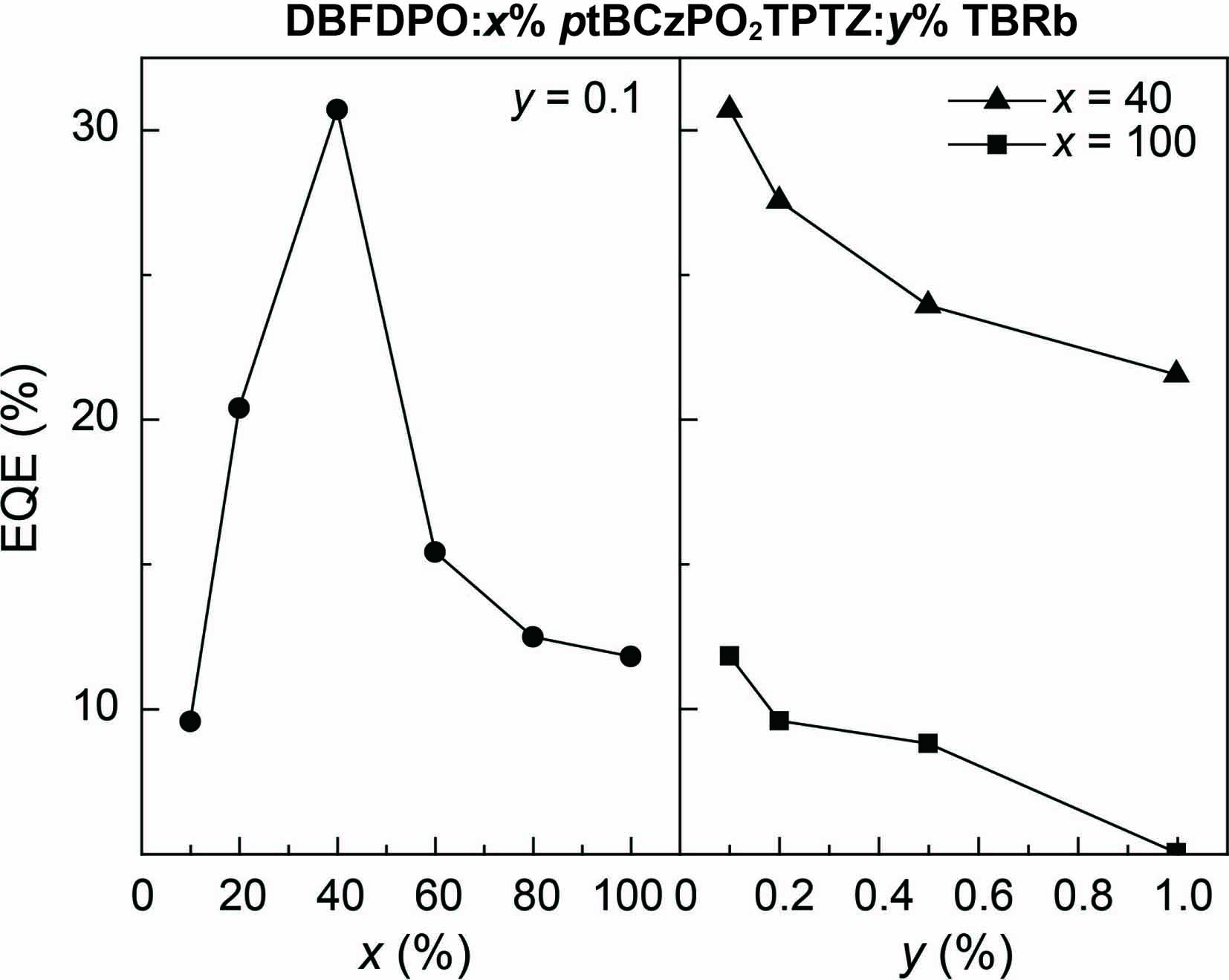


Fig. S18.

Dependence of the maximum EQE values on doping concentrations of DBFDPO:*x*% *p*tBCzPO_2_TPTZ:*y*% TBRb based devices.

Table S1.

Photophysical properties of *p*tBCzPO_2_TPTZ, 2CzPN and DMAC-DPS based films.

| Doped films | *φ*_PL_^[a]^  (%) | *φ*_PF_^[b]^  (%) | *φ*_DF_^[c]^  (%) | *k*_PF_^[d]^  (10^7^ s^-1^) | *k*_DF_^[e]^  (10^4^ s^-1^) | *k*_ISC_^[f]^  (10^6^ s^-1^) | *k*_RISC_^[g]^  (10^4^ s^-1^) | $k_{r}^{S}$^[h]^  (10^6^ s^-1^) | $k_{nr}^{S}$^[i]^  (10^6^ s^-1^) | $k_{nr}^{T}$^[j]^  (10^4^ s^-1^) | *φ*_ISC_^[k]^  (%) | *φ*_RISC_^[l]^  (%) |
| --- | --- | --- | --- | --- | --- | --- | --- | --- | --- | --- | --- | --- |
| DBFDPO:40% *p*tBCzPO_2_TPTZ:0.1% TBRb | 99 | 51 | 48 | 3.11 | 3.03 | 14.98 | 5.84 | 16.01 | 0.16 | 0.03 | 48 | 99 |
| DBFDPO:40% 2CzPN:0.1% TBRb | 47 | 25 | 22 | 1.08 | 0.97 | 5.09 | 1.84 | 2.68 | 3.03 | 0.51 | 47 | 78 |
| DBFDPO:40% DMAC-DPS:0.1% TBRb | 10 | 5 | 5 | 0.22 | 1.10 | 1.17 | 2.35 | 0.10 | 0.94 | 0.99 | 53 | 70 |
| *p*tBCzPO2TPTZ:0.1% TBRb | 41 | 25 | 16 | 1.35 | 1.83 | 5.37 | 3.04 | 3.33 | 4.80 | 1.08 | 40 | 74 |
| DBFDPO:40% *p*tBCzPO_2_TPTZ | 94 | 42 | 52 | 2.84 | 3.59 | 15.61 | 7.98 | 12.01 | 0.77 | 0.22 | 55 | 97 |
| DBFDPO:40% 2CzPN | 70 | 37 | 33 | 1.68 | 0.75 | 7.97 | 1.42 | 6.21 | 2.66 | 0.22 | 47 | 86 |
| DBFDPO:40% DMAC-DPS | 65 | 39 | 26 | 1.86 | 3.61 | 7.32 | 5.95 | 7.32 | 3.94 | 1.26 | 39 | 82 |

^[a]^ Absolute PL quantum yield measured with integrating spheres; quantum efficiencies of prompt fluorescence (PF)^[b]^ and prompt fluorescence (DF)^[c]^; Rate constants of PF^[d]^, DF^[e]^, intersystem crossing (ISC)^[f]^, reverse intersystem crossing (RISC)^[g]^, singlet radiation^[h]^ and nonradiation^[i]^, and triplet nonradiation^[j]^; ^[k]^ ISC efficiency; ^[l]^ RISC efficiency.

Table S2.

Device performance of DBFDPO:x% blue TADF emitter:y% TBRb *p*tBCzPO_2_TPTZ, 2CzPN and based white OLEDs.

| Blue TADF emitter | *x* | *y* | *V*^[b]^  (V) | *L*_max_  (cd m^-2^) | *η*_max_^[c]^ | | | CIE (x, y)/CCT (K)^[d]^ |
| --- | --- | --- | --- | --- | --- | --- | --- | --- |
|  |  |  |  |  | *η*_CE_ (cd A^-1^) | *η*_PE_ (lm W^-1^) | *η*_EQE_ (%) |  |
| *p*tBCzPO_2_TPTZ | 10 | 0.1 | 3.4, 5.0, 7.5 | 9675 | 22.0, 21.3, 15.9 | 20.0, 13.2, 6.7 | 9.6, 9.2, 6.9 | (0.21, 0.35)/13493 |
|  | 20 |  | 2.9, 4.0, 5.5 | 24310 | 53.5, 52.0, 43.7 | 56.5, 40.1, 24.5 | 20.4, 19.8, 16.3 | (0.28, 0.41)/6897 |
|  | 40 |  | 2.5, 3.3, 4.1 | 40105 | 96.1, 93.5, 86.3 | 120.2, 91.1, 65.3 | 30.7, 30.0, 26.9 | (0.30, 0.42)/6225 |
|  | 60 |  | 2.5, 3.4, 4.7 | 47220 | 48.3, 46.1, 38.9 | 60.7, 43.0, 25.0 | 15.5, 14.7, 12.5 | (0.34, 0.47)/5316 |
|  | 80 |  | 2.5, 3.4, 4.9 | 38260 | 39.1, 37.9, 32.0 | 49.2, 36.0, 20.5 | 12.5, 12.1, 10.2 | (0.36, 0.49)/4930 |
|  | 100 |  | 2.5, 3.5, 5.0 | 34836 | 38.3, 37.0, 33.7 | 48.8, 33.1, 21.8 | 11.8, 11.4, 10.4 | (0.33, 0.49)/5486 |
|  | 40 | 0 | 2.6, 3.8, 5.2 | 9757 | 45.4, 44.1, 35.6 | 54.9, 38.1, 22.7 | 19.8, 19.0, 16.2 | (0.17, 0.36)/18427 |
|  |  | 0.2 | 2.5, 3.4, 4.3 | 47339 | 92.0, 89.0, 81.2 | 112.8, 83.2, 61.3 | 27.6, 26.7, 24.3 | (0.34, 0.47)/5301 |
|  |  | 0.5 | 2.5, 3.3, 4.2 | 29948 | 82.7, 80.8, 76.4 | 101.8, 75.7, 57.8 | 24.0, 23.4, 22.2 | (0.36, 0.48)/4914 |
|  |  | 1.0 | 2.5, 3.3, 4.1 | 32189 | 77.1, 73.4, 67.0 | 95.3, 69.1, 50.8 | 21.6, 20.6, 18.7 | (0.42, 0.48)/3921 |
|  | 100 | 0.2 | 2.5, 3.4, 4.6 | 40590 | 33.1, 31.5,27.1 | 42.8, 28.9, 16.9 | 9.6, 9.1, 7.6 | (0.37, 0.53)/4763 |
|  |  | 0.5 | 2.5, 3.6, 5.2 | 21946 | 31.5, 29.1, 23.7 | 41.2, 24.5, 13.5 | 8.8, 8.0, 6.4 | (0.38, 0.53)/4670 |
|  |  | 1.0 | 2.6, 4.0, 5.7 | 17622 | 18.0, 17.7, 15.7 | 21.7, 14.1, 8.9 | 5.1, 4.9, 4.4 | (0.44, 0.52)/3772 |
| 2CzPN | 10 | 0.1 | 3.5, 5.5, 8.5 | 12760 | 18.6, 17.6, 9.9 | 16.9, 10.1, 3.6 | 7.2, 6.8, 3.8 | (0.21, 0.36)/12815 |
|  | 20 |  | 3.0, 4.5, 7.0 | 15400 | 27.7, 24.7, 13.8 | 29.4, 17.8, 6.3 | 10.3, 9.1, 5.1 | (0.22, 0.39)/10459 |
|  | 40 |  | 2.7, 3.8, 5.6 | 26060 | 36.2, 35.5, 24.4 | 43.2, 30.7, 13.6 | 12.4, 12.2, 8.4 | (0.25, 0.46)/7733 |
|  | 60 |  | 2.5, 3.8, 5.5 | 29530 | 19.8, 19.2, 16.4 | 24.6, 16.3, 9.4 | 6.4, 6.2, 5.3 | (0.29, 0.50)/6394 |
| DMAC-DPS | 10 | 0.1 | 3.0, 6.0, 9.0 | 4243 | 11.2, 8.6, 6.3 | 11.2, 4.5, 2.2 | 4.9, 3.7, 2.8 | (0.19, 0.33)/18521 |
|  | 20 |  | 2.7, 4.7, 7.1 | 3566 | 12.0, 10.1, 6.9 | 14.2, 6.8, 3.1 | 4.3, 3.6, 2.5 | (0.26, 0.43)/7922 |
|  | 40 |  | 2.6, 4.4, 6.6 | 4228 | 9.1, 8.6, 6.4 | 11.0, 6.7, 3.0 | 3.0, 2.9, 2.1 | (0.32, 0.50)/5829 |
|  | 60 |  | 2.6, 4.3, 6.5 | 4585 | 7.7, 7.6, 6.5 | 9.3, 5.9, 3.0 | 2.4, 2.4, 2.0 | (0.35, 0.52)/5175 |
| *p*tBCzPO_2_TPTZ^[a]^ | 10 | 0.1 | 3.0, 4.9, 7.0 | 3863 | 32.5, 24.1, 14.4 | 34.6, 15.3, 6.5 | 11.3, 8.4, 5.0 | (0.25, 0.42)/8492 |
|  | 20 |  | 2.7, 4.1, 5.8 | 13650 | 40.2, 37.8, 28.9 | 47.5, 29.2, 16.0 | 13.8, 12.9, 9.8 | (0.25, 0.44)/8069 |
|  | 40 |  | 2.6, 3.8, 5.4 | 27440 | 56.0, 53.1, 47.2 | 67.7, 42.4, 28.2 | 21.3, 20.1, 17.8 | (0.25, 0.46)/7789 |
|  | 60 |  | 2.8, 4.6, 6.7 | 21020 | 57.9, 55.6, 42.9 | 66.2, 41.1, 20.0 | 19.5, 18.7, 14.5 | (0.26, 0.49)/7251 |
|  | 80 |  | 2.7, 4.0, 5.7 | 39050 | 40.0, 38.7, 36.0 | 47.4, 29.5, 20.0 | 12.7, 12.2, 11.4 | (0.29, 0.49)/6537 |

^[a]^ In DPEPO matrix; ^[b]^ at 1, 100 and 1000 cd m^-2^; ^[c]^ for maximum and 100 and 1000 cd m^-2^; ^[d]^ CIE coordinates and CCT at 1000 cd m^-2^.

Table S3.

EL performance of representative TADF involved white OLEDs.

| Device structure | *V*^[a]^  (V) | *L*_Max_  (cd m^-2^) | *η*_max_^[b]^ | | | CIE (x, y)^[c]^ | Ref. |
| --- | --- | --- | --- | --- | --- | --- | --- |
|  |  |  | *η*_CE_ (cd A^-1^) | *η*_PE_ (lm W^-1^) | *η*_EQE_ (%) |  |  |
| ITO\|HATCN\|TAPC:HATCN\|TAPC\|CBP:FDQPXZ\|  DPEPO:DMAC-DPS\|DPEPO\|BmPyPB:Li_2_CO_3_\|Li_2_CO_3_\|Al | 2.6, 2.8, 3.2 | < 30000 | 51.3, 46.5\|31.9 | 59.6, 52.4, 31.7 | 20.5, 18.8, 13.0 | 0.33, 0.41 | (*3*) |
| ITO\|TAPC\|*m*CP\|TspiroS-TRZ:DTPA-ADO\|DPEPO\|  TmPyPb\|LiF\|Al | 3.6, 4.6, 5.8 | < 7000 | 53.0, 47.3, 33.3 | 43.7, 32.3, 19.4 | 22.8, 20.5, 14.6 | 0.28, 0.38 | (*4*) |
| ITO\|HATCN\|NPB\|TCTA\|32aICTRZ:DACz-TAZTRZ\|  mCPBC:TDBA-DI\|CzPhPy\|p-bPPhen\|LiF\|Al | 2.7, 3.7, 4.3 | <20000 | - | 57.7, -, 51.2 | 30.7-, 30.3 | 0.31, 0.37 | (*5*) |
| ITO\|MoO_3_\|*m*CP\|246DBFTPO:DMAC-DPS:4CzTPNBu\|  246DBFTPO\|LiF\|Al | 2.7, 3.9, 5.2 | 37010 | 66.0, 64.6, 60.1 | 76.7, 52.2, 36.1 | 21.9, 21.3, 19.8 | 0.40, 0.48 | (*6*) |
| ITO\|MoO_3_\|*m*CP\|DBFDPO:SSFAPO:4CzTPNBu\|  DBFDPO\|LiF\|Al | 2.9, 3.9, 5.1 | 14375 | 77.6, 74.0, 64.5 | 82.6, 58.8, 40.9 | 25.1, 23.9, 20.8 | 0.41, 0.49 | (*7*) |
| ITO\|TAPC\|TCTA\|*m*CP\|DBFCz-Trz:BPPZ-DPXZ\|  TmPyPb\|LiF\|Al | 2.6, 3.2, 3.7 | <40000 | 95.4, 82.6, 70.1 | 99.9, 80.0, 58.0 | 32.8, 28.4, 24.1 | 0.41, 0.46 | (*8*) |
| MoO_3_\|*m*CP\|*m*CP:*Dp*PBITPO:DMAC-DPS:4CzTPNBu\|  *p*DPBITPO\|LiF\|Al | 2.7, 4.2, 6.7 | 37000 | 93.1, 89.8, 84.3 | 108.2, 68.4, 39.5 | 32.7, 31.5, 29.6 | 0.44, 0.47 | (*9*) |
| ITO\|PEDOT:PSS\|CzAcSF:PCzAQC0.5\|DPEPO\|  TmPyPB\|Liq\|Al | 5.0, 6.6, 8.9 | 3287 | 25.4, -, - | 15.6, -, - | 22.4, -, 13.0 | 0.52, 0.38 | (*10*) |
| ITO\|PEDOT:PSS\|CBP\|OPDPO\|2CzTPEPCz\|  TPBI\|Mg:Ag | 6.1, -, 8.2 | 12310 | 45.9, -, 30.6 | 18.0, -, 11.5 | 20.8, -, 13.7 | 0.45, 0.44 | (*11*) |
| ITO\|HAT-CN\|α-NPD\|Tris-PCz\|mCBP\|mCBP:5Cz-TRZ:  DCJTB\|PPF:5Cz-TRZ:TBRb \|CF3-TRZ\|Liq:BPPB\|Liq\|Al | < 3.0, 3.2, 3.9 | < 20000 | 54.0, 47.5, 38.0 | - | 21.8, 20.0, 16.5 | 0.43, 0.45 | (*12*) |
| ITO\|HATCN\|NPB\|SFBCz\|SFBCz:SFTRZ:TCzBN:  TBRb\|SFTRZ\|DPyPA\|LiF\|Al | 2.5, -, 3.9 | < 40000 | - | 78.0, -, 48.4 | 21.7, -, 21.4 | 0.40, 0.51 | (*13*) |
| ITO\|HATCN\|TAPC\|TCTA:DBP\|CBP:BDMAC-XT:  4CzTPNBu\|TCTA\|PPF:DCP-BP-DPAC\|PPF\|TmPyPB\|LiF\|Al | 2.6, 2.9, 3.4 | 13530 | 53.3, -, 34.5 | 64.4, -, 30.9 | 23.0, 20.1, 14.1 | 0.32, 0.41 | (*14*) |
| ITO\|HAT-CN\|NPB\|mCBP\|2tCz2CzBN:1PXZ-BP\|  SF3-TRZ\|Alq_3_:Liq\|Liq\|Al | 2.7, 4.6, 6.7 | 24010 | - | 50.0/17.8/7.5 | 23.2/9.8/6.1 | 0.40, 0.45 | (*15*) |
| ITO\|HAT-CN\|NPB\|mCBP\|mCBP:3Ph_2_CzCzBN:TBRb\|  SF3-TRZ\|Alq_3_:Liq\|Liq\|Al | 4.1, 5.8, 7.7 | 87690 | 54.9, 48.0 | - | 20.9, 20.0, 18.2 | 0.31, 0.41 | (*16*) |
| ITO\|HATCN\|NPB\|TCTA\|mCPBC:5TCzBN:TBRb\|  CzPhPy\|DPPyA\|LiF\|Al | 2.7, -, - | < 30000 | - | 52.2, -, 29.1 | 19.6, 18.0, 15.4 | 0.33, 0.45 | (*17*) |
| ITO\|*m*CP\|DBFDPO:*p*tBCzPO_2_TPTZ:TBRb\|  *p*TPOTZ\|LiF\|Al | 2.5, 3.3, 4.1 | 40105 | 96.1, 93.5, 86.3 | 120.2, 91.1, 65.3 | 30.7, 30.0, 26.9 | 0.30, 0.42 | This work |

^[a]^ At 1, 100 and 1000 cd m^-2^; ^[b]^ for maximum and 100 and 1000 cd m^-2^; ^[c]^ CIE coordinates and CRI at 1000 cd m^-2^.

Device performances of hyperfluorescence white OLEDs were marked with gray background color.

References

1. C. Li, C. Duan, C. Han, H. Xu, Secondary Acceptor Optimization for Full-Exciton Radiation: Toward Sky-Blue Thermally Activated Delayed Fluorescence Diodes with External Quantum Efficiency of ≈30%. *Adv. Mater.* **30**, 1804228 (2018).

2. J. Jia, L. Zhu, Y. Wei, Z. Wu, H. Xu, D. Ding, R. Chen, D. Ma, W. Huang, Triazine-phosphine oxide electron transporter for ultralow-voltage-driven sky blue PHOLEDs. *J. Mater. Chem. C* **3**, 4890-4902 (2015).

3. Z. Wu, Y. Liu, L. Yu, C. Zhao, D. Yang, X. Qiao, J. Chen, C. Yang, H. Kleemann, K. Leo, D. Ma, Strategic-tuning of radiative excitons for efficient and stable fluorescent white organic light-emitting diodes. *Nat. Commun.* **10**, 2380 (2019).

4. W. Li, B. Li, X. Cai, L. Gan, Z. Xu, W. Li, K. Liu, D. Chen, S.-J. Su, Tri-Spiral Donor for High Efficiency and Versatile Blue Thermally Activated Delayed Fluorescence Materials. *Angew. Chem. Int. Ed.* **58**, 11301-11305 (2019).

5. C. Zhang, D. Zhang, Z. Bin, Z. Liu, Y. Zhang, H. Lee, J. H. Kwon, L. Duan, Color-Tunable All-Fluorescent White Organic Light-Emitting Diodes with a High External Quantum Efficiency Over 30% and Extended Device Lifetime. *Adv. Mater.* **33**, 2103102 (2021).

6. J. Zhang, C. Han, F. Du, C. Duan, Y. Wei, H. Xu, High-Power-Efficiency White Thermally Activated Delayed Fluorescence Diodes Based on Selectively Optimized Intermolecular Interactions. *Adv. Funct. Mater.* **30**, 2005165 (2020).

7. Y. Li, Z. Li, J. Zhang, C. Han, C. Duan, H. Xu, Manipulating Complementarity of Binary White Thermally Activated Delayed Fluorescence Systems for 100% Exciton Harvesting in OLEDs. *Adv. Funct. Mater.* **33**, 2011169 (2021).

8. J.-X. Chen, K. Wang, Y.-F. Xiao, C. Cao, J.-H. Tan, H. Wang, X.-C. Fan, J. Yu, F.-X. Geng, X.-H. Zhang, C.-S. Lee, Thermally Activated Delayed Fluorescence Warm White Organic Light Emitting Devices with External Quantum Efficiencies Over 30%. *Adv. Funct. Mater.* **31**, 2101647 (2021).

9. C. Han, R. Du, H. Xu, S. Han, P. Ma, J. Bian, C. Duan, Y. Wei, M. Sun, X. Liu, W. Huang, Ladder-like energy-relaying exciplex enables 100% internal quantum efficiency of white TADF-based diodes in a single emissive layer. *Nat. Commun.* **12**, 3640 (2021).

10. T. Wang, K. Li, B. Yao, Y. Chen, H. Zhan, Z. Xie, G. Xie, X. Yi, Y. Cheng, Rigidity and Polymerization Amplified Red Thermally Activated Delayed Fluorescence Polymers for Constructing Red and Single‐Emissive‐Layer White OLEDs. *Adv. Funct. Mater.* **30**, 2002493 (2020).

11. J. Zhao, Z. Yang, X. Chen, Z. Xie, T. Liu, Z. Chi, Z. Yang, Y. Zhang, M. P. Aldred, Z. Chi, Efficient triplet harvesting in fluorescence–TADF hybrid warm-white organic light-emitting diodes with a fully non-doped device configuration. *J. Mater. Chem. C* **6**, 4257-4264 (2018).

12. L.-S. Cui, A. J. Gillett, S.-F. Zhang, H. Ye, Y. Liu, X.-K. Chen, Z.-S. Lin, E. W. Evans, W. K. Myers, T. K. Ronson, H. Nakanotani, S. Reineke, J.-L. Bredas, C. Adachi, R. H. Friend, Fast spin-flip enables efficient and stable organic electroluminescence from charge-transfer states. *Nat. Photonics* **14**, 636-642 (2020).

13. C. Zhang, Y. Lu, Z. Liu, Y. Zhang, X. Wang, D. Zhang, L. Duan, A π–D and π–A Exciplex-Forming Host for High-Efficiency and Long-Lifetime Single-Emissive-Layer Fluorescent White Organic Light-Emitting Diodes. *Advanced Materials* **32**, 2004040 (2020).

14. H. Liu, J. Chen, Y. Fu, Z. Zhao, B. Z. Tang, Achieving High Electroluminescence Efficiency and High Color Rendering Index for All-Fluorescent White OLEDs Based on an Out-of-Phase Sensitizing System. *Adv. Funct. Mater.* **31**, 2103273 (2021).

15. F.-M. Xie, S.-J. Zou, Y. Li, L.-Y. Lu, R. Yang, X.-Y. Zeng, G.-H. Zhang, J. Chen, J.-X. Tang, Management of Delayed Fluorophor-Sensitized Exciton Harvesting for Stable and Efficient All-Fluorescent White Organic Light-Emitting Diodes. *ACS Appl. Mater. Inter.* **12**, 16736-16742 (2020).

16. S. J. Zou, F. M. Xie, Y. Q. Li, Y. Z. Shi, Y. Shen, Z. G. Ma, J. D. Chen, H. X. Wei, X. H. Zhang, J. X. Tang, Partial energy transfer from blue TADF sensitizer to orange fluorescent dopant for prolonging device lifetime. *Materials Today Energy* **21**, 100745 (2021).

17. P. Wei, D. Zhang, L. Duan, Modulation of Förster and Dexter Interactions in Single-Emissive-Layer All-Fluorescent WOLEDs for Improved Efficiency and Extended Lifetime. *Adv. Funct. Mater.* **30**, 1907083 (2020).
